# Epithelial-interleukin-1 inhibits collagen formation by airway fibroblasts: Implications for asthma

**DOI:** 10.1038/s41598-020-65567-z

**Published:** 2020-05-26

**Authors:** Emmanuel T. Osei, Leila B. Mostaço-Guidolin, Aileen Hsieh, Stephanie M. Warner, May AL-Fouadi, Mary Wang, Darren J. Cole, Geoffrey N. Maksym, Teal S. Hallstrand, Wim Timens, Corry-Anke Brandsma, Irene H. Heijink, Tillie-Louise. Hackett

**Affiliations:** 1 Centre for Heart Lung Innovation, University of British Columbia (UBC), Vancouver, B.C. Canada; 2 Department of Anesthesiology, Pharmacology and Therapeutics, University of British Columbia, Vancouver, B.C. Canada; 3 University of Groningen, University Medical Center Groningen, Department of Pathology and Medical Biology, Groningen, the Netherlands; 4 University of Groningen, GRIAC (Groningen Research Institute of Asthma and COPD), University Medical Center Groningen, Groningen, the Netherlands; 5 University of Groningen, University Medical Center Groningen, Department of Pulmonology, Groningen, the Netherlands; 6 University of Washington Medical Center, Washington, U.S.A.; 7 Dalhousie University, School of Biomedical Engineering, Halifax, Nova Scotia Canada; 8 Present Address: Department of Systems and Computer Engineering, Faculty of Engineering and Design, Carleton University, Ottawa, Canada

**Keywords:** Cell signalling, Cellular imaging, Cytoskeleton, Mechanisms of disease, Cells, Cell biology

## Abstract

In asthma, the airway epithelium has an impaired capacity to differentiate and plays a key role in the development of airway inflammation and remodeling through mediator release. The study objective was to investigate the release of (IL)-1 family members from primary airway epithelial-cells during differentiation, and how they affect primary airway fibroblast (PAF)-induced inflammation, extracellular matrix (ECM) production, and collagen I remodeling. The release of IL-1α/β and IL-33 during airway epithelial differentiation was assessed over 20-days using air-liquid interface cultures. The effect of IL-1 family cytokines on airway fibroblasts grown on collagen-coated well-plates and 3-dimensional collagen gels was assessed by measurement of inflammatory mediators and ECM proteins by ELISA and western blot, as well as collagen fiber formation using non-linear optical microscopy after 24-hours. The production of IL-1α is elevated in undifferentiated asthmatic-PAECs compared to controls. IL-1α/β induced fibroblast pro-inflammatory responses (CXCL8/IL-8, IL-6, TSLP, GM-CSF) and suppressed ECM-production (collagen, fibronectin, periostin) and the cell’s ability to repair and remodel fibrillar collagen I via LOX, LOXL1 and LOXL2 activity, as confirmed by inhibition with β-aminopropionitrile. These data support a role for epithelial-derived-IL-1 in the dysregulated repair of the asthmatic-EMTU and provides new insights into the contribution of airway fibroblasts in inflammation and airway remodeling in asthma.

## Introduction

Asthma is a common, chronic respiratory disease characterized by airway inflammation and remodeling which leads to episodes of shortness of breath^1^. Several longitudinal studies of children to adulthood have shown that current asthma treatments manage symptoms, but do not improve airflow obstruction caused by airway remodeling^2–4^. The structural changes that lead to airway remodeling include: altered epithelial barrier, sub-epithelial fibrosis with an altered deposition and composition of the extracellular matrix (ECM), increased smooth muscle mass, goblet and mucous gland hyperplasia, angiogenesis, and inflammation^5^. The airway epithelium forms the first structural and immunological barrier to the inhaled environment within the context of the epithelial mesenchymal tropic unit (EMTU)^6^. In asthma, the airway epithelium has been shown to play a key role in the development of airway inflammation and remodeling, through signaling which recruits and activates immune cells, and resident tissue mesenchymal cells^6–8^. Several studies have demonstrated that in asthma, the airway epithelium *in vivo* and when grown in air-liquid interface culture has decreased adherens junction^9,10^, and tight-junction formation^11,12^ an impaired capacity to differentiate with increased numbers of basal cells^10^ and an exuberant inflammatory response to environmental stimuli^9,11,13^.

In asthmatics, in response to environmental triggers such as house dust mite, interleukin (IL)-1 is released as an immune mediator or damage associated molecular pattern (DAMP) together with other master-cytokines including; thymic stromal lymphopoietin (TSLP) and granulocyte monocyte-colony stimulating factor (GM-CSF), resulting in eosinophil recruitment, immunoglobulin (Ig)-E switching, and the release of T_H_2 inflammatory mediators (IL-4, IL-9, IL-13)^14,15^. Further, studies involving asthma patients, allergen mouse models and *in vitro* cell models, have shown IL-1 signaling to be directly involved in various aspects of airway remodeling including: smooth muscle activation and airway hyperresponsiveness^16,17^, chronic mucus hypersecretion^18^ and abnormal production of ECM proteins such as fibronectin^19^.

Within the EMTU, fibroblasts are the primary mesenchymal cells involved in tissue homeostasis through the production and repair of ECM proteins, that provide the structural and biochemical support essential for cell bioactivity and survival^20,21^. Many previous studies have demonstrated that collagen I, is the most abundant ECM protein deposited within the asthmatic airways^22^. The use of non-linear optical microscopy has recently highlighted that the fibrillar collagen I present in asthmatic airways is not only increased but also highly disorganized^23^.

We have demonstrated using a novel co-culture model that lung epithelial-derived-IL-1α is an essential mediator that modulates inflammatory mediator release and ECM protein expression by lung parenchymal fibroblasts^24^. In the present study, we hypothesized that lack of airway epithelial differentiation may play an essential role in driving airway fibroblast mediated inflammation and ECM remodeling within the asthmatic airway EMTU. Using cells derived from asthmatic and non-asthmatic subjects, we assessed the expression of IL-1 family members, over a time course of 20 days of epithelial differentiation in ALI culture. We report that only IL-1α/β release was elevated during the first 5 days of airway epithelial differentiation in asthmatic derived ALI-cultures compared to non-asthmatics. Further, IL-1α/β can affect airway fibroblast-driven inflammation and collagen I formation, which has implications for airway remodeling in asthma.

## Results

### Undifferentiated asthmatic airway epithelial cells release elevated levels of IL-1

**Figure 1:**
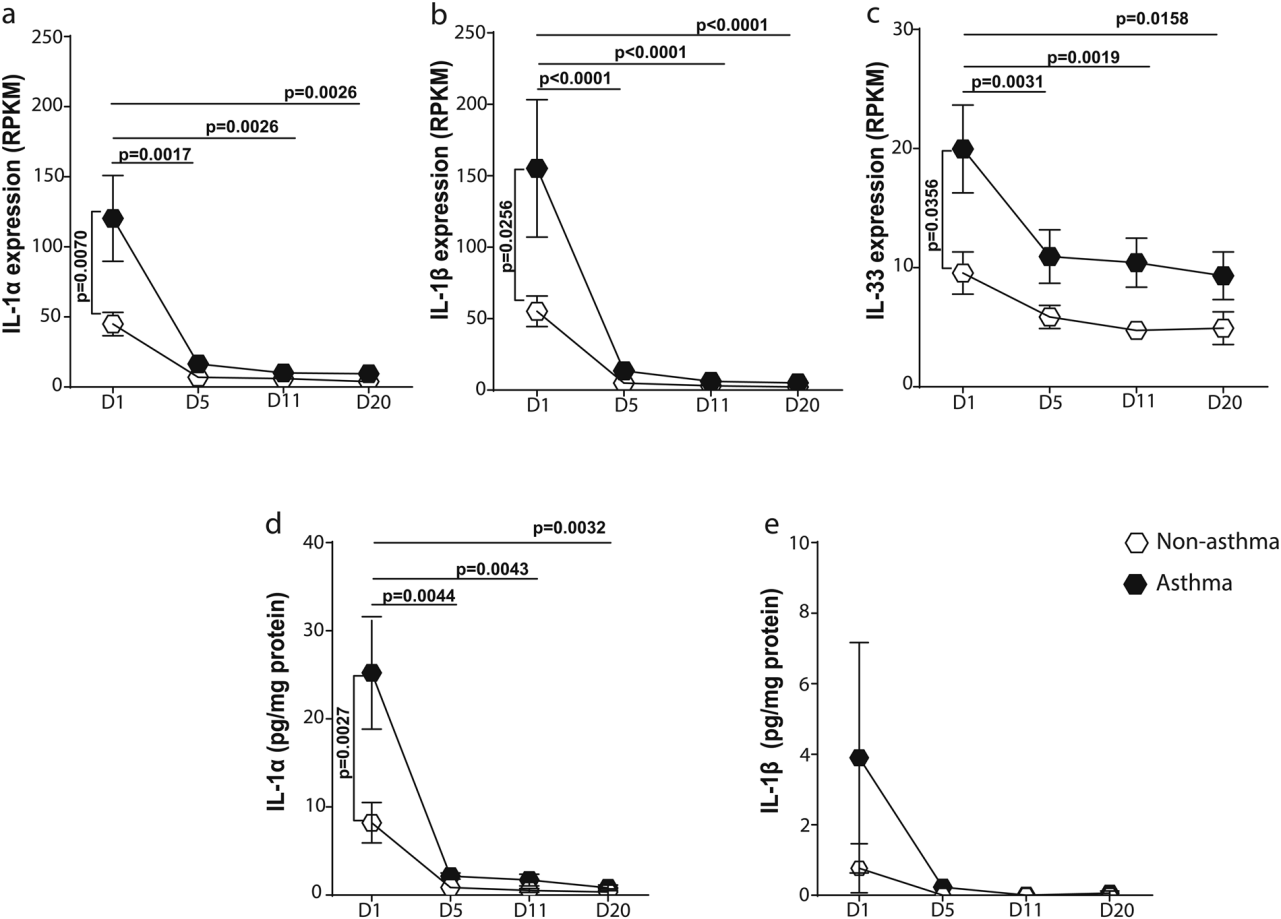
Production of IL-1 & IL-33 in differentiated air-liquid interface (ALI) cultures of primary airway epithelial cells. Primary airway epithelial cells (PAECs) from non-asthmatics (n = 5) and asthmatics (n = 10) were cultured at an air-liquid interface, RNA and supernatants were collected at Days (D) 1, 5, 11 and 20. (**a**) IL-1α (**b**) IL-1β and (**c**) IL-33 expression from PAECs are expressed as normalized to base pair reads. The concentration of (**d**) IL-1α & (**e**) IL-1β released from PAECs was measured by ELISA. Means ± SEM are shown. Exact P values indicated.

An air liquid interface (ALI) culture model was used to assess the expression of IL-1 family members during epithelial differentiation. Primary airway epithelial cells (PAECs) from asthmatics expressed greater mRNA levels of IL-1α (Fig. 1a), IL-1β (Fig. 1b), and IL-33 (Fig. 1c) when in a basal cell monolayer after transition to air-liquid-interface at day 1, compared to non-asthmatics. As PAECs polarized and differentiated into a pseudostratified epithelium (day 5 to 20 of ALI culture), the expression of IL-1 family members in asthmatic-derived PAECs decreased to the levels of non-asthmatic-derived cells (Fig. 1a–c). The mRNA expression of other IL-1 family members, IL-1RA, IL-1RII, IL-18, IL-36α and β, was not different at baseline or during epithelial differentiation (see Supplementary Fig. S1). In addition, we assessed the expression of other epithelial mediators shown to play a role in asthma. Like IL-1α, we found a similar pattern of increased expression of GM-CSF, IL-8 and transforming growth factor (TGF)-β1 in PAEC-ALI cultures at day 1 which decreased during differentiation and no changes in TSLP expression. There was no difference in the expression of these cytokines at any time point between asthmatic and non-asthmatics PAEC-ALI cultures (see Supplementary Fig. S2).

When the PAEC culture supernatant was assessed for protein release, asthmatic-PAECs secreted significantly greater levels of IL-1α when in a monolayer at day 1 compared to non-asthmatic-PAECs (Fig. 1d). Although the level of IL-1β was elevated in supernatant from asthmatic PAECs at day 1, this was not statistically significant compared to non-asthmatics (Fig. 1e). The levels of IL-33 in all samples were below the detection limit of the assay. We also assessed whether the increased expression of IL-1 was accompanied by an abnormal expression of transcription factors, NF-κB, c-JUN and c-FOS and found no significant differences in these factors (see Supplementary Fig. S3).

To confirm previous findings of decreased epithelial differentiation in asthmatic airways, we found a decrease in the percentage of ciliated cells (see Supplementary Fig. 4b) and an increase in cytokeratin (CK)-5 positive basal cells (see Supplementary Fig. 4c) in PAEC-ALI cultures from asthmatic donors compared to non-asthmatics at day 20. We found no difference in transepithelial electrical resistance of asthmatic and non-asthmatic PAEC-ALI cultures (see Supplementary Fig. S4d).

We have previously shown with lung epithelial and parenchymal fibroblast co-culture and conditioned-medium (CM) experiments that epithelial-derived IL-1α is the main mediator involved in lung parenchymal epithelial-fibroblast communication^24^. To confirm if this mediator was also involved in airway epithelial-fibroblast communication, we exposed primary airway fibroblasts (PAFs) to PAEC-CM pre-treated with the IL-1α neutralizing antibody or a broad matrix metalloproteinase (MMP) inhibitor (TAPI-2) (see Supplementary Fig. S5). Here, we found that in line with our previous study^24^, blocking IL-1α led to a complete inhibition of PAF-derived CXCL8/IL-8 confirming that epithelial-derived-IL-1α was also involved in airway epithelial-fibroblast cross-talk.

### IL-1 stimulates inflammatory mediator release from airway fibroblasts

**Figure 2:**
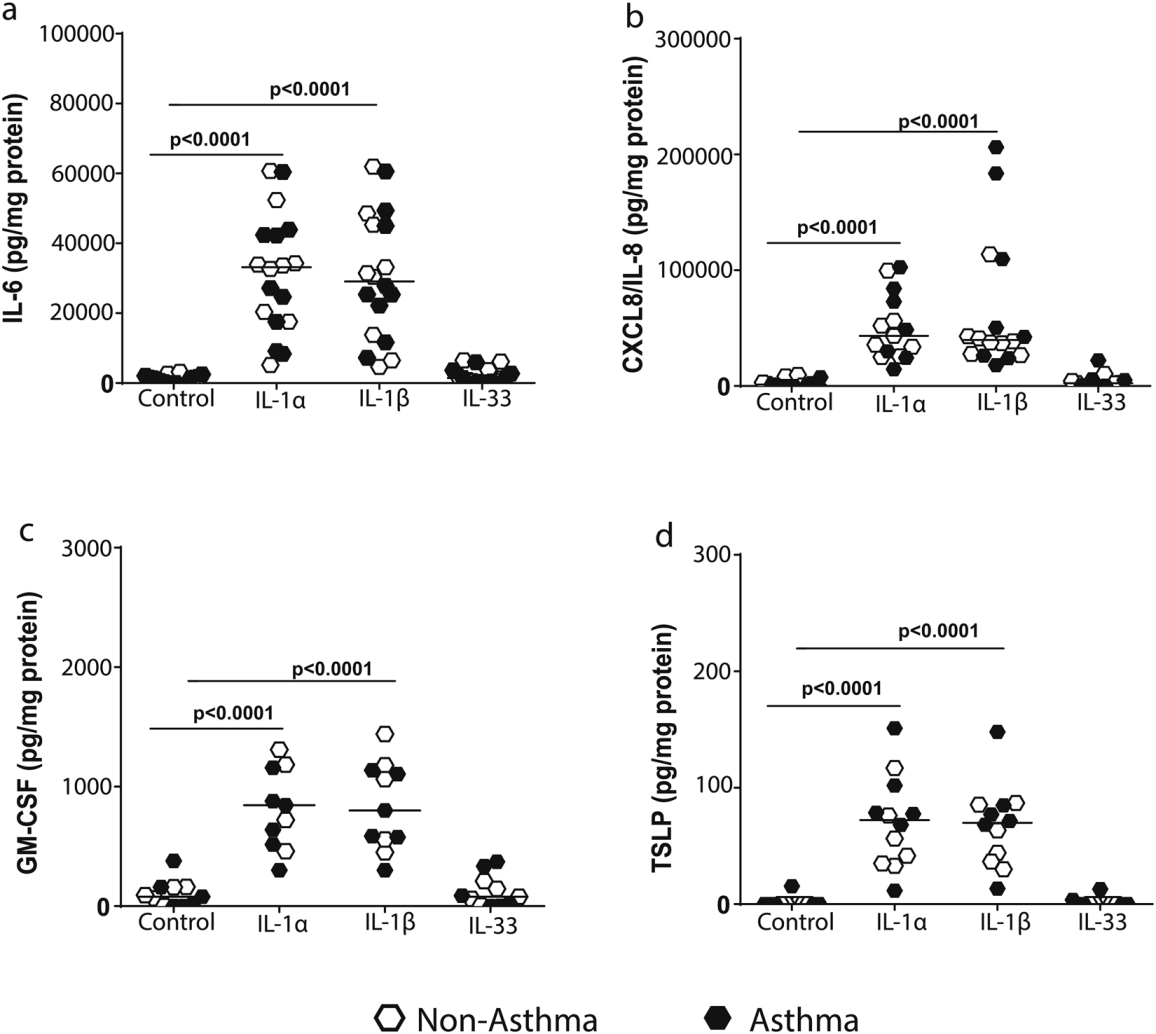
IL-1 but not IL-33 stimulates the release of inflammatory mediators from primary airway fibroblasts (PAFs). Primary airway fibroblasts from non-asthmatics (n = 5–9) and asthmatics (n = 5–9) were grown to confluence on collagen I coated plates and stimulated with or without 1 ng/ml recombinant human IL-1α, IL-1β or IL-33 for 24 hours. Concentration of (**a**) IL-6, (**b**) CXCL8/IL-8, (**c**) granulocyte-monocyte colony stimulating factor (GM-CSF) & (**d**) thymic stromal lymphopoietin (TSLP) released from primary airway fibroblasts after 24 hours. Exact P values indicated.

To determine the importance of IL-1α in airway epithelial-fibroblast crosstalk in asthma, we assessed the isolated effects of the IL-1 family members on PAFs from asthmatic and non-asthmatic donors. This allowed us to not only assess the specific effects of IL-1α but also compare this to IL-1β and IL-33 which were the other family members with increased expression in the differentiating ALI model. We did this by stimulating airway fibroblasts with a 1 ng/ml dose of recombinant IL-1α, IL-1β or IL-33 for 24 hours and then assessed the release of inflammatory mediators known to be involved in chronic inflammation, and airway hyperresponsiveness in asthma^7,21^. IL-1α and IL-1β, but not IL-33 induced a significant increase in the release of inflammatory mediators, IL-6 (Fig. 2a), CXCL8/IL-8 (Fig. 2b), GM-CSF (Fig. 2c) and TSLP (Fig. 2d) by PAFs. To ascertain the reason for no biological response by PAF’s to IL-33 stimulation, we assessed the expression of the ST2 receptor for IL-33. Here, we found that in comparison to the IL-1R1 receptor and other receptors important for fibroblast function such as the TGFRs the ST2 receptor has a very low expression in airway fibroblasts, which could explain the lack of cellular response to IL-33 we found in our experiments (see Supplementary Fig S6). There was no difference in the response of non-asthmatic and asthmatic-derived PAFs to IL-1 stimulation. Further, stimulation with IL-1α/β, or IL-33 did not affect the viability of PAFs (see Supplementary Fig. S7).

### IL-1 suppresses extracellular matrix expression by airway fibroblasts

**Figure 3:**
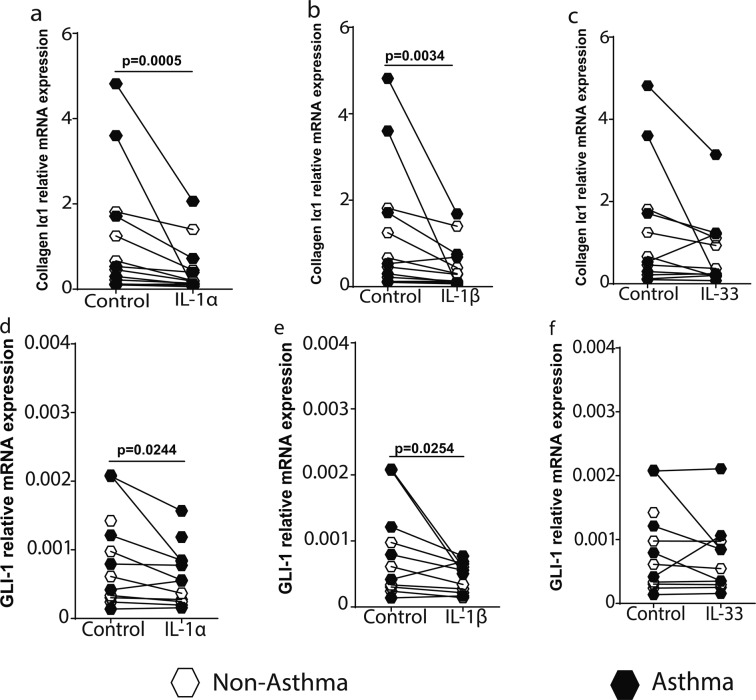
IL-1α and IL-1β induce decreased extracellular matrix protein expression in airway fibroblasts. Primary airway fibroblasts from non-asthmatics and asthmatics were grown to confluence on collagen I coated plates and stimulated with or without 1 ng/ml recombinant human IL-1α, IL-1β or IL-33 for 24 hours. The mRNA expression of Collagen Iα1 (**a–c**), and glioma-associated oncogene homolog 1 (GLI-1) (**d–f**) was assessed at 24 hours. Exact P values indicated.

Next, we assessed the effects of IL-1 on airway fibroblast-ECM expression. IL-1α and IL-1β, but not IL-33, significantly suppressed the mRNA (Fig. 3a–c) and protein (see Supplementary Fig. S8a and Fig. S9 for representative full collagen blots) expression of collagen Iα1 in PAFs, with no difference in the response between asthmatic and non-asthmatic donors. IL-1 has been shown to suppress collagen expression via down-regulation of the sonic Hedgehog (SHH) transcription factor glioma-associated oncogene homolog 1 (GLI-1) in human lung fibroblasts^25^. We found IL-1α and IL-1β, but not IL-33 down-regulated GLI-1 expression in PAFs (Fig. 3d–f). In addition to collagen Iα1, IL-1α/β, but not IL-33, also suppressed the expression of the ECM proteins, periostin and fibronectin, which are known to be altered in expression in asthma (see Supplementary Fig. S8b–g). However, IL-1 stimulation had no effect on the expression of the proteoglycan decorin (see Supplementary Fig. S8h–j), which has been shown to be involved in the spacing of collagen fibrils. There were no differences in the response of asthmatic and non-asthmatic-derived airway fibroblasts to IL-1α/β or IL-33 stimulation.

### IL-1 inhibits collagen I fiber formation and contraction by airway fibroblasts

**Figure 4:**
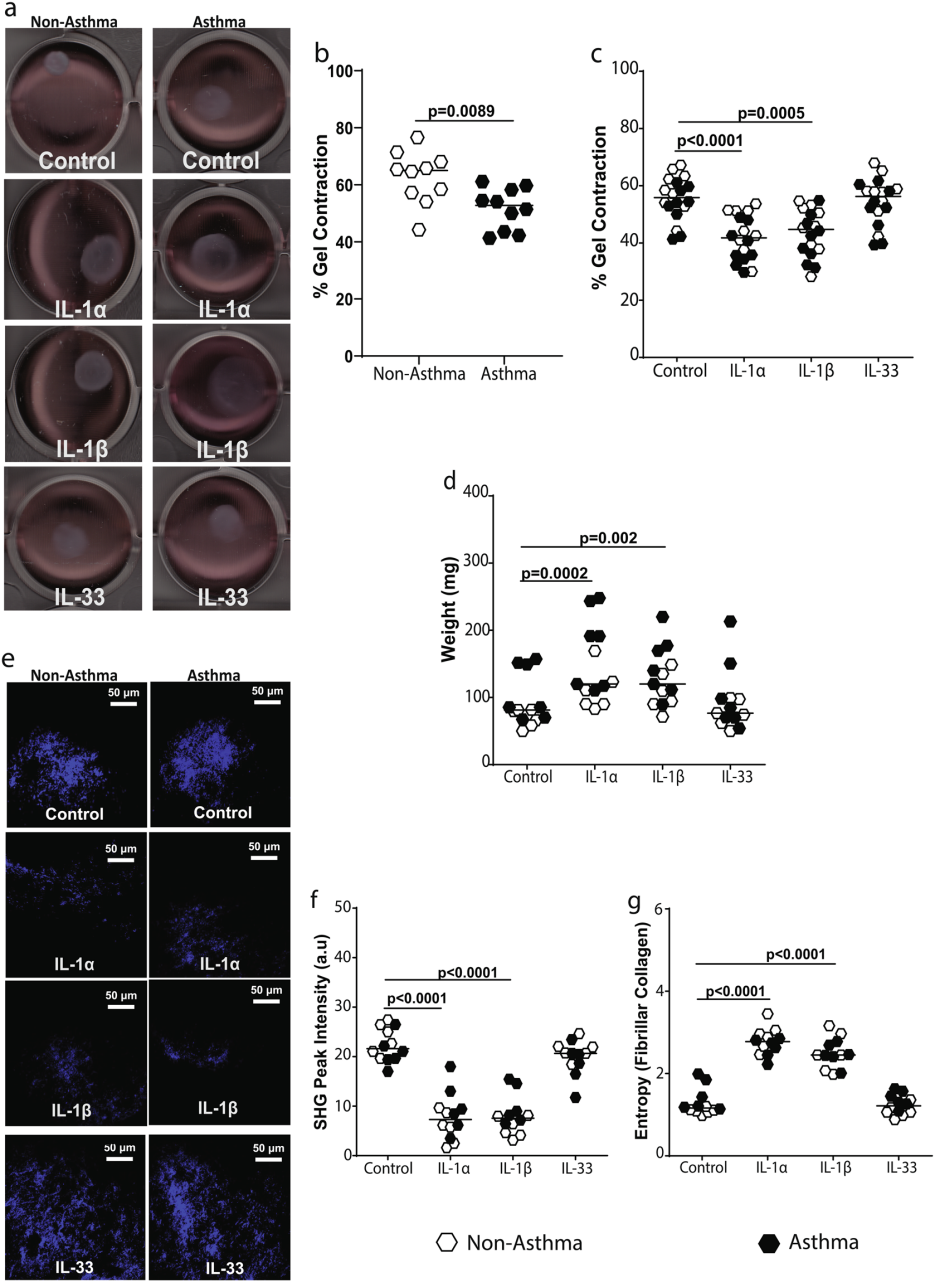
IL-1 but not IL-33 affects fibroblast collagen I gel contraction and fiber formation. Primary airway fibroblasts (PAFs) from non-asthmatics and asthmatics were grown to confluence and seeded on 3D collagen I gels in the presence or absence of 1 ng/ml IL-1α, IL-1β or IL-33 and allowed to migrated into and contract the gel for 24 hours. (**a**) Representative gel contraction images after the 24 hour stimulation of non-asthmatic and asthmatic PAF-seeded gels with 1 ng/ml IL-1α, IL-1β or IL-33, (**b**) Percentage gel contraction comparing control non-stimulated rates of non-asthmatic and asthmatic -derived PAFs, (**c**) Percentage gel contraction after stimulation with IL-1α, IL-1β and IL-33, (**d**) semi-dry weight of contracted gels after stimulation with IL-1α, IL-1β and IL-33. (**e**) Representative images of fibrillar collagen taken with second harmonic generation (SHG) microscopy in gels, (**f**) SHG peak intensity of fibrillar collagen. (**g**) Entropy of collagen fibers, calculated using texturel analysis of SHG images. Representative images are 20X magnification and scale bars are provided. Exact P values indicated. SHG images are pseudo-coloured, showing fibrillar collagen in blue (un-labeled).

It is well known in literature that collagen deposition is an important feature of asthmatic airway remodeling^5,22^. Importantly, we have recently shown that airway fibroblasts are not only essential for collagen deposition but also its repair and remodeling in the airways^23^. To assess the ability of airway fibroblasts to repair and remodel fibrillar collagen I, a free-floating hydrolyzed rat-tail collagen I gel contraction assay was used. Airway fibroblasts were seeded on top of the gel and allowed to migrate through and contract the gel for 24 hours. Representative images of contraction of PAF-seeded collagen gels after IL-1α/β and IL-33 stimulation compared to non-stimulated controls are shown in Fig. 4a. We have previously shown that asthmatic-derived fibroblasts are defective in collagen I gel contraction in a 72 hour collagen gel contraction model^23^, and this was confirmed in this study (Fig. 4b) in our 24 hour model. In response to IL-1α and IL-1β, but not IL-33, PAFs were inhibited in the remodeling and contraction of the hydrolyzed collagen I compared to non-stimulated control conditions (Fig. 4c). There was no difference in the response of non-asthmatic and asthmatic airway fibroblasts to IL-1α and IL-1β stimulation. During gel-contraction, there is extrusion of water from collagen gels due to matrix compaction by fibroblasts which causes gel-weight to decrease^26^. In validation of the inhibition of contraction by IL-1α and IL-1β, the collagen I gel weight did not decrease when stimulated with IL-1α and IL-1β, compared to IL-33 treatment and non-stimulated controls (Fig. 4d).

To determine if IL-1α and IL-1β inhibited collagen I gel contraction by airway fibroblasts due to defective collagen fiber formation, we analyzed the structure of collagen fibers using second harmonic generation microscopy (SHG) (Fig. 4e) as previously described^27^. Collagen I gels seeded with airway fibroblasts and treated with IL-1α/β had a significantly lower SHG peak intensity for collagen compared to non-stimulated controls, indicating cells were unable to form fibrillar collagen (Fig. 4f). Using texture analysis, we assessed the degree of disorder of fibrillar collagen fibers, reported as entropy, within the collagen I gels^28^. We found that collagen I gels treated with IL-1α and IL-1β, had a greater measure of entropy, indicating more disorganization of collagen fibers compared to IL-33 and non-stimulated control conditions (Fig. 4g). We found no differences in the formation of fibrillar collagen in response to IL-1α/β treatment between non-asthmatic and asthmatic derived airway fibroblasts.

### IL-1 alters the cytoskeleton of airway fibroblasts and correlates with reduced fibrillar collagen formation

**Figure 5:**
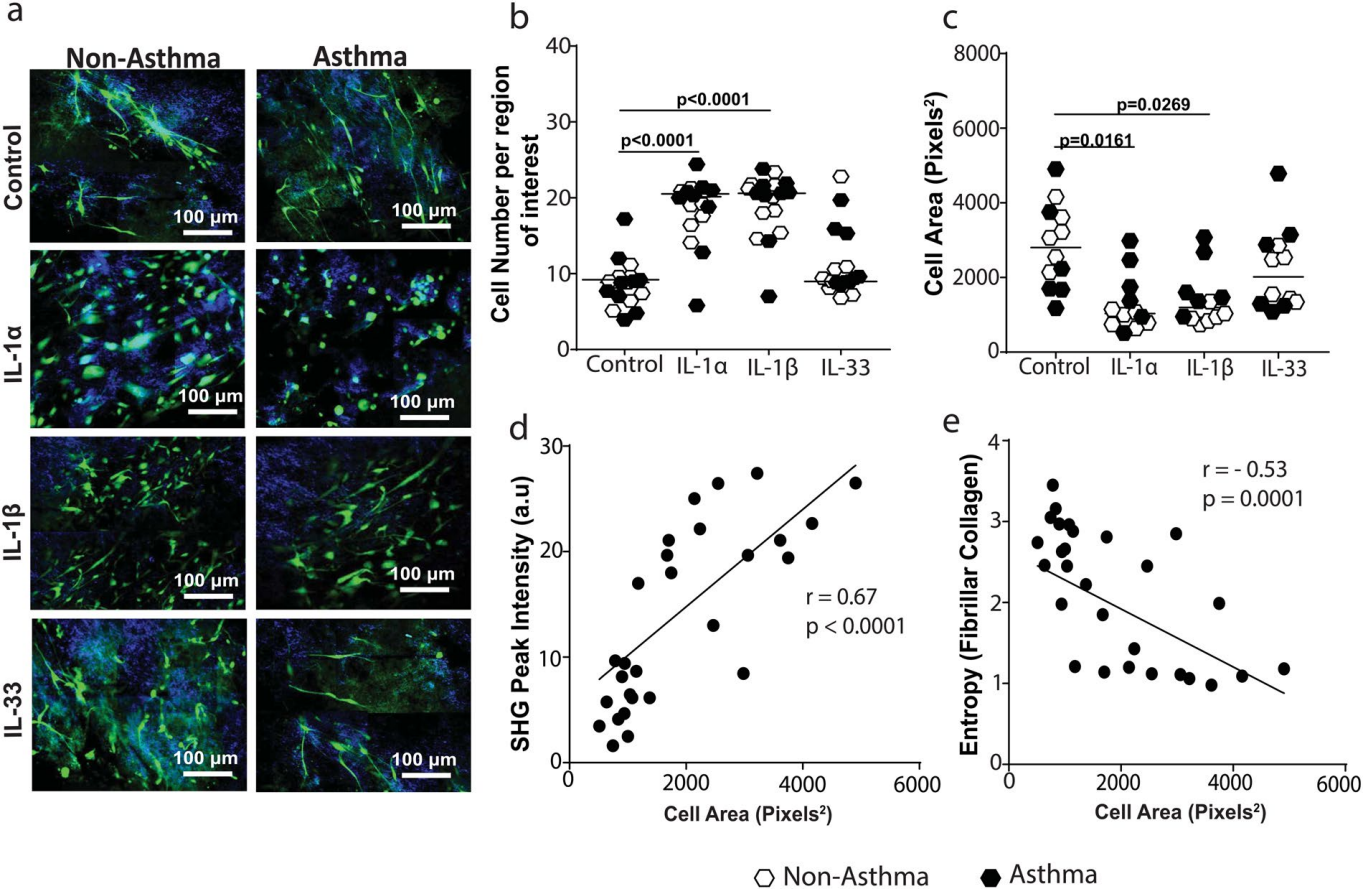
Interleukin-1 alters fibroblast interaction with collagen I. Primary airway fibroblasts from non-asthmatics and asthmatics were grown to confluence and seeded on collagen I gels in the presence or absence of 1 ng/ml IL-1α, IL-1β or IL-33 and allowed to migrate through and contract for 24 hours. Collagen I gels were then stained with DAPI to localize cell nuclei and Phalloidin 488 for F-actin in the seeded fibroblasts. (**a**) Representative composite images of co-localized un-labeled second harmonic generation images (SHG, shown in blue) and two-photon excited fluorescence labeled with DAPI and phalloidin (TPEF, shown in green) of fibrillar collagen and fibroblasts respectively in gels from non-asthmatic and asthmatic PAF’s (**b**) Average number of cells per region of interest, (**c**) Cell area of fibroblasts seeded in collagen I gels after contraction. (**d**) Correlation of fibroblast cell area with SHG peak intensity of fibrillar collagen, **(e**) Correlation of fibroblast cell area with collagen fiber entropy. Representative images are 20X magnification and scale bars are provided. Exact P values indicated.

To assess if IL-1 influenced the interaction of airway fibroblasts with collagen I fibers, collagen I gels were stained with Phalloidin to image cytoskeletal F-actin within cells (Fig. 5a). We observed that although IL-1α and IL-1β, caused an increased proliferation of airway fibroblasts (Fig. 5b), both inhibited the formation of dendritic extensions within airway fibroblasts resulting in cell rounding and a reduction in total cell area compared to IL-33 and control non-stimulated conditions (Fig. 5c). The effects of IL-1α/β on cell rounding and proliferation were not different between asthmatic and non-asthmatics and assessing PAFs for E-cadherin to determine if morphological changes was indicative of mesenchymal-to-epithelial transition showed no staining (see Supplementary Fig. S10). Interestingly, the reduced fibroblast cell area strongly correlated with the decreased SHG signal intensity (Fig. 5d) and increased disorganization (entropy, Fig. 5e) of fibrillar collagen in the gels.

### IL-1 controls fibroblast-mediated collagen remodeling via lysyl oxidase regulation

**Figure 6:**
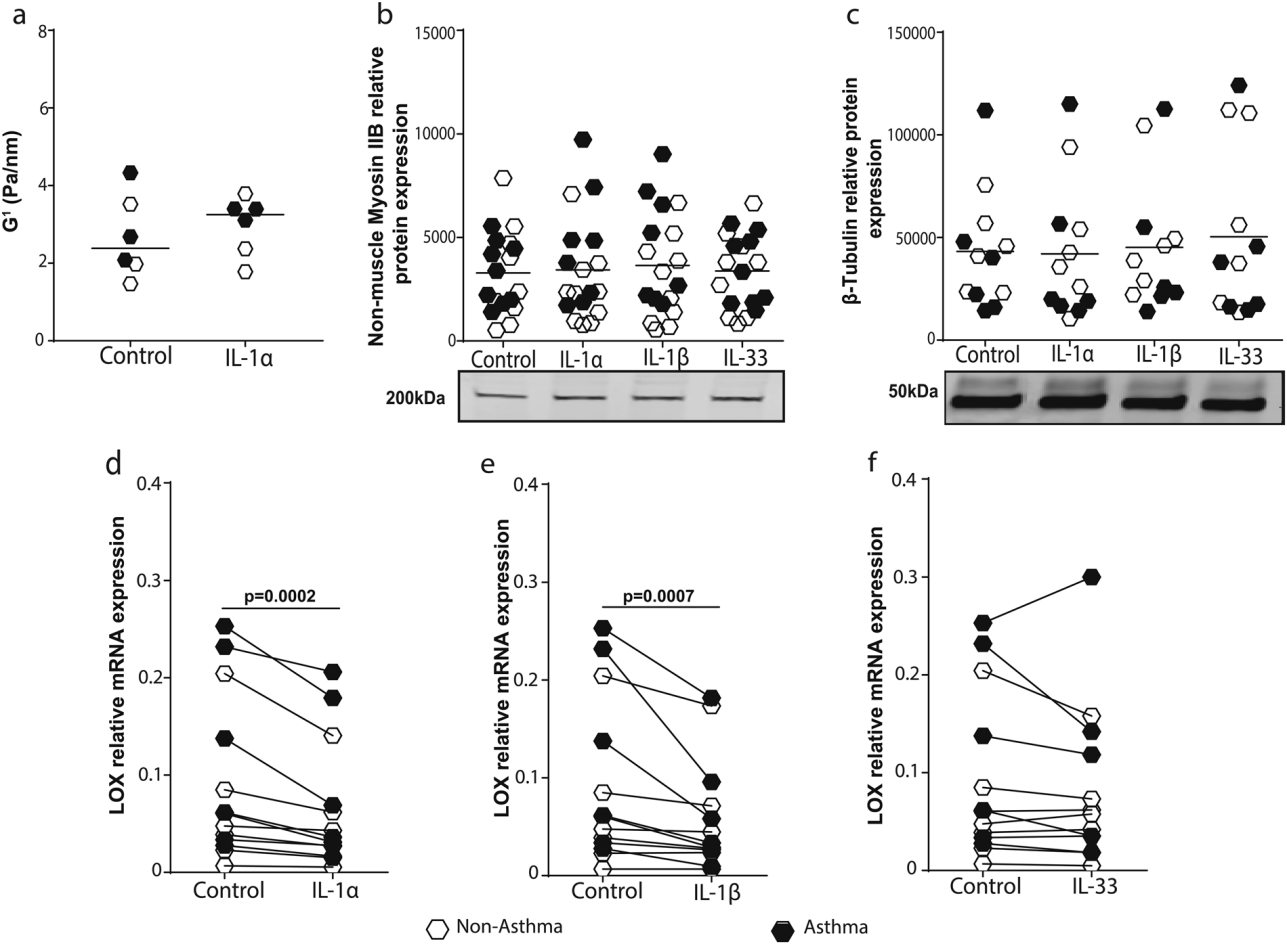
IL-1 down-regulates the expression of lysyl oxidase (LOX) in airway fibroblasts. Primary airway fibroblasts (PAFs) from non-asthmatics and asthmatics were seeded on 96 well plates in the presence of 1 ng/ml IL-1α, (**a**) Optical magnetic twisting cytometry was then used to measure cell stiffness presented as G^1^ (Pa/nm) (n = 6). PAFs were grown to confluence on collagen I coated 6-well plates and stimulated with or without 1 ng/ml recombinant IL-1α/β or IL-33 for 24 hours. Relative protein expression and representative blot images of (**b**) non muscle myosin IIB and (**c**) β-tubulin. mRNA expression of LOX after stimulation with (**d**) IL-1α, (**e**) IL-1β and **(f**) IL-33. Exact P values indicated.

Next, we investigated the mechanism by which IL-1 inhibits fibrillar collagen remodeling by airway fibroblasts. First, we assessed the effect of IL-1 on the rheology of airway fibroblasts using optical magnetic twisting cytometry (OMTC)^29^, and found no effect on cell stiffness (Fig. 6a, P = 0.31). We then assessed the protein expression of cytoskeletal proteins; non-muscle myosin IIB (NMIIB) (Fig. 6b), and β-tubulin (Fig. 6c) (see Supplementary Fig. S11 for representative images of full western blots) important for fibroblast movement and interaction with fibrillar collagen and found no effect of IL-1.

**Figure 7:**
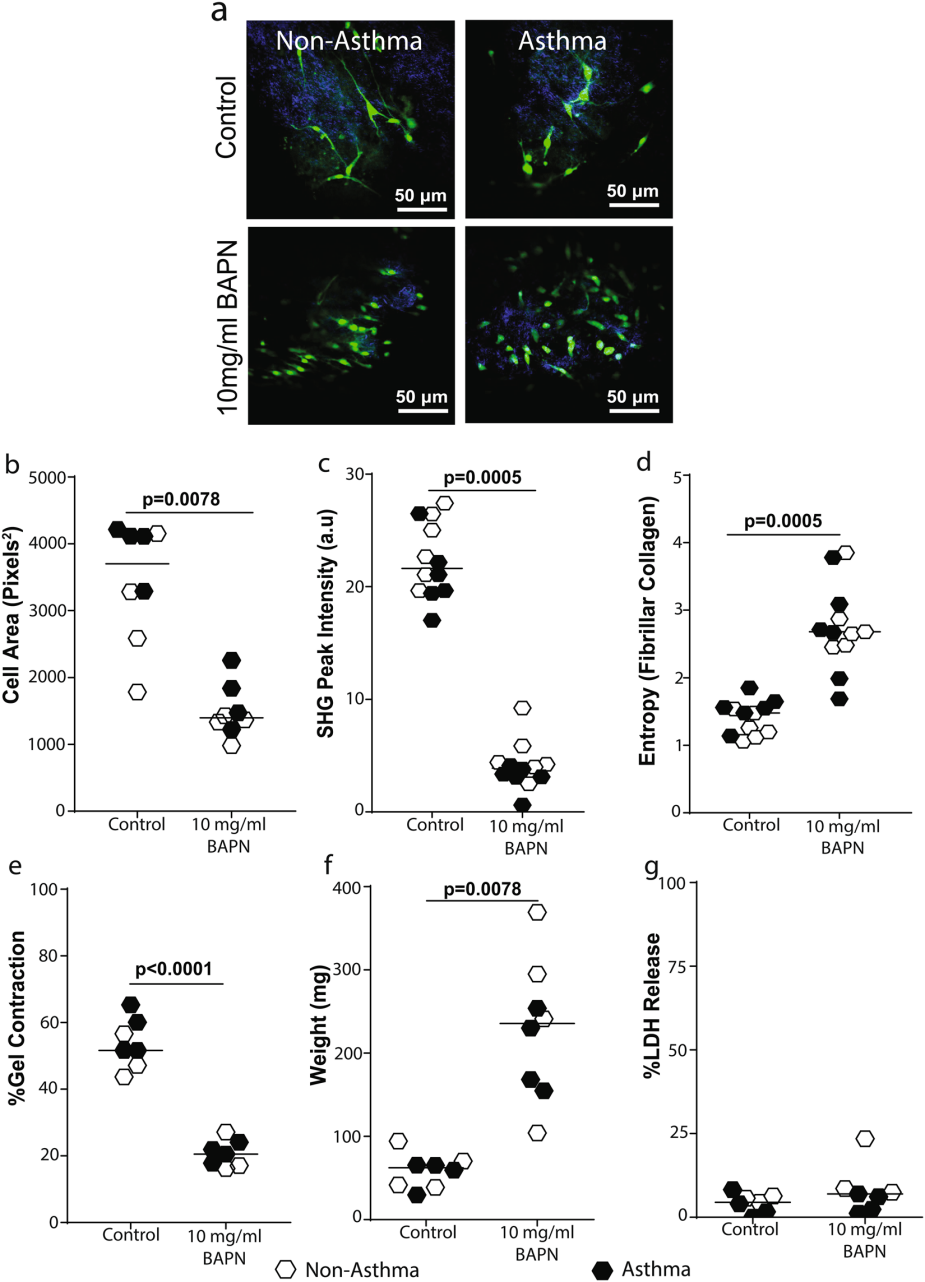
Lysyl oxidase activity is essential for fibroblast contraction of collagen I gels. Primary airway fibroblasts from non-asthmatics and asthmatics (n = 4–6) were grown to confluence and seeded on collagen I gels and allowed to contract and migrate through the gel for 24 hours in the presence or absence of 10 mg/ml β-aminopropionitrile which inhibits lysyl oxidase activity. Collagen I gels were then stained with DAPI and Phalloidin 488 for F-actin in the seeded fibroblasts. (**a**) Representative composite images of co-localized un-labeled second harmonic generation images (SHG, shown in blue) and two-photon excited fluorescence labeled with DAPI and phalloidin staining (TPEF, shown in green) of fibrillar collagen and fibroblasts respectively, (**b**) Cell area of fibroblasts seeded in collagen I gels after contraction. (**c**) Second harmoni generation (SHG) peak intensity of fibrillar collagen and (**d**) entropy score for collagen fibers calculated using texturel analysis of second harmonic generation images (**e**) Percentage gel contraction of collagen I gels. (**f**) Semi-dry weight of contracted gels. (**g**) Primary airway fibroblasts (PAFs) were grown to confluence on collagen I coated plates and stimulated with or without 10 mg/ml BAPN. Percentage lactate dehydrogenase (LDH) released from cells after 24 hours. Representative images are 20X magnification and scale bars are provided. Exact P values indicated.

Lysyl oxidase (LOX) is an important enzyme known to regulate fibroblast morphology and remodeling of fibrillar collagen^30^. We found IL-1α and IL-1β, but not IL-33, induced significant down-regulation of LOX in PAFs (Fig. 6d–f). LOX-like enzymes 1 and 2 have also been shown to play a role in ECM remodeling by PAFs^31^. In response to IL-1α and IL-1β stimulation, the expression of both enzymes were also down-regulated compared to basal unstimulated controls (see Supplementary Fig. S12). To determine if the LOX enzymes are responsible for the IL-1-induced inhibition of collagen I remodeling by PAFs, cells were treated with the broad LOX inhibitor β-aminopropionitrile (BAPN). BAPN treatment induced loss of dendritic extensions and cell rounding (Fig. 7a), leading to a decreased cell area (Fig. 7b), a decreased fibrillar collagen intensity (Fig. 7c), increased fibrillar collagen I disorganization (entropy, Fig. 7d), and an almost complete inhibition of collagen I gel contraction by primary airway fibroblasts (Fig. 7e,f), comparable to IL-1α and IL-1β treatment. Cell death analysis indicated BAPN stimulation did not affect cell viability (Fig. 7g).

## Discussion

We report that, the production of IL-1α is elevated in undifferentiated asthmatic airway epithelial ALI-cultures compared to that of healthy non-asthmatics. Furthermore, we show that IL-1α and its family member IL-1β are important for regulating airway fibroblast pro-inflammatory responses (CXCL8/IL-8, IL-6, TSLP, GM-CSF), ECM production (collagen, fibronectin, periostin) and their ability to repair and remodel fibrillar collagen I via LOX activity. These data support a role for IL-1 in the dysregulated repair of the asthmatic EMTU.

The airway epithelium is a constitutive source of IL-1, storing it primarily as a plasma membrane-associated pro-form^32^. When the epithelium is damaged, IL-1 is released as an alarmin to initiate inflammation and repair^14,33^. In this study, we used an ALI-culture model to assess the release of IL-1 family members during epithelial differentiation which has been shown to be altered within the asthmatic airways^10–12^. Data from asthmatic patients, animal models and *in vitro* studies have previously shown increased IL-1 concentrations in asthmatics compared to non-asthmatics when exposed to allergens such as house dust mite^14,15^. However, this is the first report showing elevated IL-1α-release in asthmatic airway epithelial cells during differentiation. Specifically, we found higher IL-1α expression (mRNA) and release (protein) in days 1–5 of differentiation in asthmatic -derived PAEC-ALI cultures compared to non-asthmatic controls, which then declined by day-20 of differentiation. This early release of IL-1α from airway epithelial cells is in line with acute damage models of the mouse epithelium *in vivo* that have demonstrated release of IL-1within the first hour of damage, peaking between the first 12 to 24 hours of exposure, and then switching off^33^. Further, we demonstrate that IL-1α alone was elevated in asthmatic compared to non-asthmatic PAEC-ALI cultures during differentiation, compared to other IL-1 family members and key epithelial mediators shown previously to play a role in asthma (IL-8, GM-CSF, TSLP, and TGF-β1). As the airway epithelium is exposed daily to inhaled allergens^11,34^ and the airway epithelium has been shown to be less differentiated in asthmatics^10^, we propose this could lead to an environment of persistent and exaggerated IL-1α release in asthmatic airways.

In asthma, the airway epithelium has been shown to play a key role in the development of airway inflammation and remodeling, through signaling which recruits and activates immune cells, and resident tissue mesenchymal cells^6–8^. In this study, we demonstrate that IL-1α/β induce airway fibroblasts to release pro-inflammatory cytokines including IL-6, CXCL8/IL-8, TSLP and GM-CSF. These cytokines are known to be vital for allergic sensitization, smooth muscle cell-hypercontractility and airway hyperresponsiveness as well as eosinophilia, mast cell activation and T_H_2-driven inflammation in asthma^7,21^. By the use of co-cultures and conditioned medium studies, we and others have shown that epithelial-derived IL-1α regulates parenchymal lung fibroblast-mediated inflammation^24,35^. In the present study, we confirmed using conditioned medium experiments that airway epithelial-derived IL-1α was also the main mediator involved in airway epithelial-fibroblast communication.

While we demonstrate that IL-1α is the most prominent IL-1 family member released from undifferentiated airway epithelium, in all experiments, exogenous IL-1α and IL-1β, but not IL-33 were able to affect the function of airway-derived fibroblasts. This is most likely due to the fact that IL-1α and IL-1β both bind and signal through the same IL-1 receptor (IL-1R)1, whereas IL-33 signals through the ST2 receptor^32^. While the ST2 receptor is reported to be expressed on mesenchymal cells, we show that airway fibroblasts have undetectable levels of expression (similar to E-cadherin) in comparison to the more highly expressed receptors, IL-1R and TGFβR. Which is in line with previous reports that, IL-33 is known to primarily affect immune cells including T and B cells, mast cells and basophils instead of mesenchymal cells in asthma^36^.

As part of the critical epithelial-fibroblast interaction needed for tissue homeostasis, the lung epithelium is known to regulate fibroblast-ECM expression and myofibroblast differentiation to either suppress or increase ECM production during tissue repair^37–39^. There are different epithelial mediators including IL-1^24^, prostaglandins^38^, and MMPs^40^ that are able to suppress fibroblast-ECM production. We previously reported that airway epithelial-fibroblast interactions did not involve prostaglandin derivatives^24^ and MMP family members (present study). Here we demonstrate IL-1α/β stimulation can suppress airway-fibroblast expression of ECM proteins including collagen Iα1, periostin and fibronectin. This supports the role of the epithelium in regulating fibroblast-ECM expression within the entire lung as we and others have previously demonstrated a role of IL-1 in modulating ECM expression in parenchymal lung fibroblasts^24,38,39^. Although IL-1 is a classical inflammatory mediator, it is known to regulate lung fibroblast-collagen expression via the SHH pathway^41^. In agreement with this, we demonstrate that IL-1 down-regulates the SHH transcription factor GLI1, which has been shown to be involved in fibroblast activation and the expression of ECM molecules, including collagen I^41^.

In addition to fibroblast-ECM expression, we also examined for the first time, the potential effect of airway epithelial-IL-1 on collagen remodeling by airway fibroblasts. Fibroblasts in connective tissues under resting conditions are normally organized into a dendritic network^42^. In relaxed collagen gels, fibroblasts form dendritic extensions, with neuronal-like appearances, microtubule cores and actin-rich tips, reminiscent of fibroblasts under homeostatic conditions *in vivo*^43^. These dendritic extensions enable fibroblasts to spread and interact with collagen fibrils, resulting in integrin-dependent mechanical interactions^43^. In our 24 hour experiments, we found asthmatic-derived airway fibroblasts are defective at fibrillar collagen remodeling, which replicates our previous work using a 72 hour model^23^. In addition to this, IL-1α/IL-1β further inhibited collagen I contraction, fiber formation and formation of dendritic extensions in treated fibroblast-seeded gels while causing a disordered fibrillar collagen matrix. Further, we found a strong correlation between IL-1-induced reduction in fibroblast cell shape and dendritic extensions with decreased fibrillar collagen formation and increased fiber disorganization. This points to a possible mechanism whereby within the normal EMTU, IL-1 acts to prevent excessive ECM deposition during repair, however in the asthmatic EMTU the exaggerated release of epithelial-IL-1 may drive defective fibrillar collagen disorganization in the airways. This corroborates our recent data where we showed that fibrillar collagen in asthmatic airways is highly disorganized and may add to a microenvironment that is inappropriate for repair^23^. Defective fibrillar collagen formation may be important for airway remodeling in asthma as small modifications in the structure and organization of ECM proteins such as collagen I can modify their bioactive regulation on various cells through pathogenetic mechanisms such as increased oxidative stress^44^.

As to the mechanism by which IL-1 affects the contraction and repair of collagen I, we found IL-1 had no effects on airway fibroblast-cell stiffness as well as the expression of non-muscle myosin (NM)IIB and β-tubulin which are mesenchymal markers involved in fibroblast movement within collagen^45^. The cell rounding observed was ruled out to be due to mesenchymal-epithelial transition as we found no expression of E-cadherin in fibroblasts. We also found that IL-1 had no effect on airway fibroblast decorin expression which is an important proteoglycan involved in the packaging of collagen fibrils. LOX and its family members LOXL1 and LOXL2 are important enzymes for fibroblast-cross-linking of collagen^31^ as well as the regulation of cell motility and actin polymerization in cancer cells^30^. We found that IL-1 stimulation caused a down-regulation of LOX, LOXL1 and LOXL2 expression by airway fibroblasts. To confirm the mechanism by which IL-1 would affect collagen repair via LOX, LOXL1 or LOXL2, we show that inhibition of these LOX enzymes with BAPN, a broad spectrum LOX inhibitor, inhibited collagen I contraction, fiber organization and fibroblast-dendritic extensions similar to the effects of IL-1 stimulation. These data indicate that IL-1 regulates the ability of airway fibroblasts to repair and remodel collagen I by targeting the expression of LOX enzymes. This is line with work by Mia and colleagues, who showed IL-1 inhibits TGF-β induced myofibroblast differentiation in lung fibroblasts by down-regulating LOX expression and causing the decreased expression of ECM proteins such as collagen I^25^. Further work with use of specific LOX inhibitors when available will be required to determine which specific LOX enzyme or if all are essential for this response.

This study has some limitations that must be acknowledged. We chose to study epithelial differentiation in an ALI model, as this allowed us to study low levels of released mediators from a single population of cells within the same cell state. This model did not allow us to look at how the epithelium may respond to epithelial denudation, resulting in the migration, proliferation and differentiation of cells. However, the release of IL-1 from the airway epithelium in monolayer wounding models has previously been established and characterized in several studies within the literature^14,46,47^. While we studied all IL-1 family members and several other key epithelial cytokines shown to play a role in asthma (GM-CSF, TSLP, IL-8, TGF-β) it is worth noting that we may have missed other additional inflammatory mediators released from the epithelium during differentiation that may influence fibroblast functions. Additionally, we used ALI cultures, conditioned medium-experiments, and exogenous stimulations of fibroblast-seeded 3D collagen gels to understand the specific effects of epithelial-derived IL-1 and its family on fibroblast repair phenotype. While free-tension 3D collagen gels are the best *in vitro* models to understand how mesenchymal cells remodel and repair their extracellular matrix (ECM) environment, they preclude co-culture experiments due to the technical challenges of growing a fully differentiated airway epithelium on top of a floating collagen gel. However, it is important to note that we used primary epithelial cells and fibroblast throughout this project to drive biological significance. Lastly, while we assessed the potential effect of increased epithelial-IL-1 production on airway fibroblasts, this does not rule out the contributions of other major cellular sources of IL-1 in the lungs such as macrophages^48^. Future studies using *in vitro* co-culture models with inflammatory cells such as macrophages may help elucidate other potential mediators involved.

To date, a few clinical trials have sort to use the IL-1 receptor antagonist, Canakinumab to treat asthma^49^. While these studies have not yielded clear positive outcomes, it is important to note that these trials have lacked adequate endpoints to aid the assessment of their impact on airway remodeling in asthma^47^. In addition, preclinical data for these trials have been based on animal models that differ in physiology to humans and 2D *in vitro* systems which lack the complexity to provide a clear picture on all aspects of IL-1 involvement in disease pathogenesis. The current study points to a role for IL-1α and IL-1β in both inflammation and collagen disorganization that may contribute to airway remodeling. Although it is important to target chronic inflammation in asthma as the majority of clinical trials have done, it is clear that the assessment of airway remodeling as a clinical endpoint in trials is also vital^50^. Hence future therapeutic studies can focus on how to use biologics targeting the IL-1α/β pathway and the different endpoints of inflammation and airway-ECM (collagen) remodeling (and organization) to help resolve these pathologies in asthmatic airways.

In conclusion, we show that, increased IL-1α/β release from asthmatic undifferentiated airway epithelial cells could contribute to increased inflammation and abnormal collagen remodeling by airway fibroblasts in the asthmatic-EMTU. This study offers new insights into epithelial regulation of fibroblast responses within the airway EMTU, whereby IL-1 release may promote fibroblast-mediated inflammation for immune cell recruitment, and an abnormal fibroblast-repair phenotype that leads to collagen fiber disorganization, which has previously been reported in asthma^23^. The IL-1R1-IL-1 pathway has been suggested as a potential therapeutic target in asthma^51^. Our findings provide new insights on how new therapies may provide ways of modulating fibrillar collagen I remodeling within the asthmatic airway.

## Methods

### See online supplement for full methods

#### Sample collection

Primary airway epithelial cells (PAECs) were obtained via endobronchial airway brushings from asthmatic and non-asthmatic subjects as previously described^52^. Primary airway fibroblasts (PAFs) were isolated via outgrowth technique as previously described^23^. Non-asthmatic donors had no respiratory disease and asthmatic donors had a physician-diagnosis of asthma (Table 1). Human lungs not suitable for transplantation from asthmatic and healthy non-asthmatic donors were obtained from donations to the International Institute for the Advancement of Medicine (Edison, NJ) for the purpose of medical research. All samples were obtained with informed consent, the study was approved by the Providence Health Care Research Ethics board (H13–02173) at the University of British Columbia and all methods were carried out with relevant guidelines and regulations according to the declaration of Helsinki.

**Table 1:** Characteristics of asthmatics and non-asthmatics from whom primary airway epithelial cells and primary airway fibroblasts were derived.

**Epithelial Donors**		
	**Non-Asthmatics**	**Asthmatics**
Subjects	5	10
Age, mean (SD)	31.4 (15.1)	28.3 (8.2)
Female/Male	3/2	7/3
Ethnicity (Caucasian/ Hispanic / Middle-Eastern/Asian)	4/0/0/1	10/0/0/0
FEV_1_/FVC (SD)	0.9 (0)	0.7 (0.1)
Meth PC_20_	8 (0)	0.7 (0.8)
Medication		
Albuterol %	NA	50%
Advair %	NA	50%
Fibroblast Donors*		
	**Non-Asthmatics**	**Asthmatics**
Subjects	9	9
Age, mean (range)	24.3 (15.7)	20.6 (7.8)
Female/Male	2/7	5/4
Ethnicity (Caucasian/ Hispanic / Middle-Eastern/Asian)	7/1/1/0	7/2/0/0
Medication		
% Albuterol	NA	56%
% Advair	NA	11%
% Solunidrol, Prednisone, Singulair, Zyrtec, Roxycodine	NA	33%

**FEV**_**1**_**/FVC:** Forced expiratory volume in one second over forced vital capacity**. Meth PC**_**20**_**:** Concentration of methacholine needed to decrease an individual’s FEV1 by 20%. *FEV_**1**_**/FVC and Meth PC**_**20**_ values were unavailable for primary airway fibroblast donors.

#### Air-liquid interface cultures and analysis

PAECs were cultured in bronchial epithelial growth medium (BEGM) (Lonza, Walkersville, MD). At passage 2/3, PAECs were grown at air-liquid-interface (ALI) on 0.4μm polyester transwell inserts (Corning, New York city, NY) as described previously^11,53^ and samples were harvested at day 1 and after confluent cells were transitioned to ALI, 5, 11, and 20. Total RNA was harvested from PAECs using the RNeasy (Qiagen), was assessed for quantity using a NanoDrop 8000 Spectrophotometer (Thermo Fisher Scientific) and quality using the 2100 Agilent Bioanalyzer RNA6000 Nano kit (Agilent Technologies Inc., Santa Clara, USA) and, the average RNA Integrity Number (RIN) was > 9. cDNA libraries were sequenced on an Illumina HiSeq. 2000, generating 100-base paired-end reads, with on average 95 million reads per sample. Cell-culture supernatants were also harvested on the same days and samples were assessed in duplicate via the ELISA kits for IL-1α, IL-1β, IL-33, IL-6, IL-8, GM-CSF and TSLP (R&D Systems, Minneapolis) according to manufacturer instructions and the concentration (pg/ml) normalized to total protein content (pg/mg of total protein) assessed using the Pierce Rapid Gold BCA Protein Assay Kit (Thermofisher).

#### Fibroblast stimulations and collagen I gel contraction assay

At passage 3–5, PAFs were grown to confluence in DMEM10% fetal calf serum (FCS) (Invitrogen, Burlington, ON, Canada) on collagen I (Corning, New York, NY, USA) coated 6-well plates. Cells were serum-deprived overnight and stimulated with/without 1 ng/mL recombinant IL-1α/β or IL-33 for 24 hours, which was determined following dose experiments (see Supplementary Fig. S14). Cell-culture supernatant, protein-lysates and RNA were harvested for ELISAs, western blots and qRT-PCR.

Collagen I gels were made as previously described^23^. 50,000 PAFs were seeded on top and allowed to migrate into and contract the gels. Cells were stimulated with 10 mg/ml β-aminopropionitrile (BAPN) (see Supplementary Fig. S15) or 1 ng/ml IL-1α,-β or IL-33 for 24 hours. Cell-seeded gels were assessed for contraction using Image J software and gel weight using a fine balance^26^. A lactate dehydrogenase (LDH) assay (Promega, Madison, USA) to determine cell viability after cell stimulation, was done according to the manufacturer’s instructions.

#### Imaging and cellular analysis of airway fibroblasts

Collagen I gels were fixed in 4% paraformaldehyde, stained with 4’,6-diamidino-2-phenylindole (DAPI) to identify nuclei, Phalloidin (Thermo-Fisher Scientific, Waltham, USA) for F-actin, and E-cadherin using a rabbit monoclonal (DECMA-1) antibody (Abcam, ab11512), then imaged at 20x magnification using a Zeiss LSM-880 inverted confocal microscope (Carl-Zeiss, Germany), with a Coherent Chameleon Ultra II femtosecond Ti:sapphire laser (Coherent, CA)^54^. The cell area and cell numbers of PAFs within collagen gels were determined with Fiji as previously described^23^. Second Harmonic Generation microscopy (SHG) images were acquired to assess fibrillar collagen as described previously^23^. Briefly, SHG is a label-free non-linear imaging technique, widely used to visualize biological molecules such as collagen, due to their unique non-centrosymmetric structure^27,55–58^. Fibroblasts were imaged using two-photon excitation microscopy (TPEF), where the simultaneous absorption of two photons leads to the electron excitation of fluorescent molecules in the sample. Since the probability of two-photon absorption depends on the square of the intensity of the incident light, excitation occurs only in a very small volume at the focal point^59,60^.

Image pre-processing such as background correction and intensity normalization were performed using Fiji analysis toolbox and plugins. Texture analysis was performed using a custom built texture analysis toolkit in MATLAB, and entropy (a measure of the degree of disorder of the structures within an image), was calculated based on gray level cooccurrence matrix (GLCM). Texture analysis enabled the assessment of the degree of disorganization of collagen fibers within an image (entropy)^28^.

To determine cell stiffness, PAFs were seeded on 96 well-plates and measured using optical magnetic twisting cytometry (OMTC) as previously described^29^.

#### Statistics

Differences in disease-groups and time-interactions in the ALI’s were assessed using a repeated-measures 2-way ANOVA with a Sidak post-hoc test. Shapiro-Wilk and D’Agostino normality tests were performed and comparisons between unpaired and paired observations assessed using Mann-Whitney U tests and Wilcoxon signed rank tests respectively. Correlations were done with the Pearson’s test. All tests were conducted in Graphpad Prism version 8 and P < 0.05 was considered statistically significant.

## Supplementary information

Supplementary information.
