# Supplementary material for: Epithelial-interleukin-1 inhibits collagen formation by airway fibroblasts: Implications for asthma
Source: Sci Rep. 2020 May 26;10:8721. doi: 10.1038/s41598-020-65567-z (PMC7250866; doi:10.1038/s41598-020-65567-z)
Supplement: Supplementary file 1 — Supplementary information. [file 41598_2020_65567_MOESM1_ESM.pdf]

SUPPLEMENTARY INFORMATION AND DATA

Epithelial-interleukin-1 inhibits collagen formation by airway fibroblasts: Implications for asthma

***Emmanuel T. Osei**[1,2], Leila Mostaco-Guidolin[1,2], Aileen Hsieh[1], Stephanie M. Warner[1], May Fouadi[1], Mary Wang[1], Darren J. Cole[7], Geoffrey N. Maksym[7], Teal Hallstrand[6], Wim Timens[3,4], Corry-Anke Brandsma[3,4], Irene H. Heijink[3,4,5], and Tillie-Louise. Hackett[1,2]

Affiliations:

[1]University of British Columbia (UBC), Centre for Heart Lung Innovation, Vancouver, B.C., Canada

[2]Department of Anesthesiology, Pharmacology and Therapeutics, University of British Columbia, Vancouver, BC, Canada

[3]University of Groningen, University Medical Center Groningen, Department of Pathology and Medical Biology; Groningen, the Netherlands

[4]University of Groningen, GRIAC (Groningen Research Institute of Asthma and COPD), University Medical Center Groningen; Groningen, the Netherlands

[5]University of Groningen, University Medical Center Groningen, Department of Pulmonology; Groningen, the Netherlands

[6]University of Washington Medical Center, Washington, USA

[7]Dalhousie University, School of Biomedical Engineering, Halifax, Nova Scotia, Canada

*Corresponding Author: emmanuel.osei@hli.ubc.ca

Airway Fibroblast Isolation and culture

Human lungs not suitable for transplantation from asthmatic and healthy non-asthmatic donors were obtained from donations to the International Institute for the Advancement of Medicine (Edison, NJ) for the purpose medical research. Bronchi were obtained through blunt dissection of freshly obtained lungs not suitable for transplantation. The bronchi were cut into tissue fragments by dicing them into $1mm^3$ sized pieces. 5-6 pieces were then placed in 6-well tissue culture plates with 1ml of DMEM 10% FCS and incubated at 37°C in 95% air and 5% CO2. The cell culture medium was replenished every third day which helped remove any cell debris and non-adherent cells. A confluent monolayer was formed from outgrowth of fibroblasts from the bronchial rings by the seventh day which was harvested using trypsin/EDTA (Invitrogen). The cell suspensions obtained were then seeded in a culture flask in fresh DMEM 10% FCS and labeled as passage one (P1). Airway fibroblasts were then expanded by passaging and used for experiments at P1 to P3. Fibroblasts were assessed to be positive for the fibroblast-marker, vimentin[1,2].

Collagen I gel contraction assay

Collagen I gels were made according to a previously described method [3,4]. Briefly, 12 well tissue-culture plates were coated with 1% BSA (Sigma) in DMEM (Lonza) for two hours. The medium was then removed and 1 ml of 0.4 mg/ml type I Rat tail collagen (Corning) in DMEM was added and allowed to polymerize for 16 hours at 37°C. Collagen gels were carefully detached from the sides of the plate before primary airway fibroblasts (PAFs) were trypsinized and seeded on the gels at a density of 50,000 cells per well. Immediately after seeding, PAFs were treated with either control media or recombinant human 1 ng/ml IL-1α, IL-1β and IL-33 (R&D systems). Fibroblast contraction of collagen I gels after 24 hours was quantified by imaging gels before and after the experiment and extent of gel contraction was analyzed using Image J software, and measuring semi-dry weight of gels with a fine balance.

Lastly, to understand the remodeling of collagen fibers during gel contraction, gels were then fixed in 4% paraformaldehyde then stained with 4',6-diamidino-2-phenylindole (DAPI) to identify nuclei and Phalloidin 488 (Thermo Fisher Scientific, Waltham, USA) to stain for F-actin. To assess the effects of lysyl oxidase (LOX) activity on collagen I gel contraction, seeded gels were stimulated with 10 mg/ml of β-aminopropionitrile (BAPN) fumarate salt (Sigma) which is a broad inhibitor of LOX activity.

Non-Linear Optical Microscopy and Texture analysis of Collagen I gels

Second Harmonic Generation microscopy (SHG) was carried out on fixed collagen I gels using a multi-photon microscope as described previously[5]. Fibroblasts were imaged using two-photon excitation microscopy (TPEF), where the simultaneous absorption of two photons leads to the electron excitation of fluorescent molecules in the sample. Since the probability of two-photon absorption depends on the square of the intensity of the incident light, excitation occurs only in a small volume at the focal point[7,8]. The peak intensity of fibrillar collagen was expressed as arbitrary unit (au). Texture analysis using a gray level co-occurrence matrix (GLCM) was used to calculate the probability of pixels within the image occurring with a particular gray-tone, in a predetermined direction and separated by a pre-defined distance as described by Haralick and colleagues[9]. This enables us to calculate the Entropy ($\sum_{\mathrm{IJ}=0}^{\mathrm{N}-1} P_{i,j} \log_{i,j}$) of fibrillar collagen which is a measure that helps to determine the organization of collagen fibrils. Textural features including Cell Area and Cell Number in nine different randomly selected regions or interest per gel were extracted by using a Matlab custom-built texture analysis toolkit. Some functions were based on the Matlab image processing toolbox [5] as well as ImageJ's histogram analysis toolbox.

Optical Magnetic Twisting Cytometry (OMTC)

To assess the effect of IL-1 on cell mechanical properties, cell stiffness was measured using OMTC as previously described[10,11]. Briefly, primary airway fibroblasts at passage 3 were seeded on a 96 well plate with surface bound magnetic and RGD peptide coated beads and grown to confluency. Cells were then serum starved and treated with control media or recombinant IL-1α for 24 hours. The 96 well plate was then placed on the stage on an inverted microscope (DM-IRB, Leica Microsystems) equipped with an electromagnetic twisting device and a charge-coupled device camera (1,280 × 1,024 pixels, 12-bit gray scale, SensiCam, Cooke, Auburn Hills, MI). The beads were twisted with a specific torque of 56 Pa at 0.5 Hz for 30 sec, and the camera imaged the beads continuously (~200 beads at a time) at 16 frames per twisting cycle. Using an intensity centroid algorithm, the bead positions in each recorded image were automatically determined[10] and then Fourier transformation was used to extract the displacement of each bead in response to the applied torque[10-12]. Beads with erratic, irreproducible motions were not analyzed. Thus, for a given specific torque ($\tilde{\mathrm{T}}$) applied to a bead and the resultant bead displacement ($\tilde{\mathrm{D}}$), as described above, a complex

stiffness ($\tilde{G}$) of the cell was defined as the ratio of the torque to the displacement, i.e., $\tilde{G} = (\tilde{T}/\tilde{D}) = G' + iG''$, where G′ is the in-phase component or elastic stiffness, which we hereafter refer to as cell stiffness (in Pascal/nm).

ELISA

Concentrations of IL-1α, IL-1β, IL-33, released from PAECs and concentrations of CXCL8/IL-8, IL-6, thymic stromal lymphopoietin (TSLP) and granulocyte-monocyte colony stimulating factor (GM-CSF) released from PAFs were measured by ELISA (R&D Systems, Minneapolis, USA) according to the manufacturer's instructions. The range and sensitivities of the individual assay kits are reported supplementary table 1 below.

CYTOKINE	CAT NO.	RANGE OF SENSITIVITY
IL-1 α	DY200	500 – 7.81pg/ml
IL-1 β	DY201	250 – 3.91pg/ml
IL-33	DY3300B	200 – 3.13pg/ml
IL-8/CXCL8	DY208	2000 – 31.3pg/ml
IL-6	DY206	600 – 9.38pg/ml
TSLP	DY1398	2000 – 31.3pg/ml
GM-CSF	DY215	1000pg/ml – 15.6pg/ml

Gene expression analysis

Total RNA was harvested from PAECs and PAFs using the RNeasy (Qiagen), was assessed for quality and quantity using a NanoDrop 8000 Spectrophotometer (Thermo Fisher Scientific) and quality (RNA Integrity Number (RIN) > 9) using the 2100 Agilent Bioanalyzer RNA6000 Nano kit (Agilent Technologies Inc., Santa Clara, USA).

For PAECs, cDNA libraries were sequenced on an Illumina HiSeq 2000, generating 100-base paired-end reads. RNA samples were multiplexed to four samples per flowcell lane. CASAVA version 1.8.2 was used to de-multiplex samples and generate FASTQ files. The last base of each raw read was trimmed off, and reads that failed Illumina's chastity filter (i.e., the ratio of the brightest intensity to the sum of the two brightest intensities was less than 0.6 for at least

two of the first 25 cycles) were removed. Large RNA reads that passed chastity filtering were aligned to the human genome (build hg19) using TopHat2 (version 2.0.6)[13] , by setting the estimated mean and standard deviation of the insert size to -25 and 50 bases, respectively. A BED file of Ensembl Gene (ENSG) loci was constructed from Ensembl build 69 by merging the unique exons from all Ensembl Transcripts within each ENSG record into a metagene. The BED file was used to determine the number of reads aligning to each Ensembl Gene (metagene) locus with the 'coverage' utility in the BEDTools software suite (version 2.17.0)[14]. RNA abundance was normalized to the total length (in kilobases) of all exons corresponding to each Ensembl Gene and to the number of aligned reads in each sample (in millions) to obtain Reads per Kilobase per Million reads (RPKM) values.

RNA from PAFs was converted to cDNA with the High Capacity cDNA Reverse Transcription Kit (Applied Biosystems, Foster City, CA) as per the instructions of the manufacturer. qRT-PCR was then performed for specific gene expression assays (all from Life Technologies) including collagen Iα1 (Hs00264051_m1), fibronectin (Hs00365052_m1), periostin (Hs01566734_m1), glioma-associated oncogene homolog 1 (GLI-1) (Hs00942480_m1), Lysyl oxidase (Hs00942480_m1), lysyl-oxidase like 1 (Hs00935937_m1), lysyl-oxidase like 2 (Hs00158757_m1) and decorin (Hs00754870_s1) on the VIIA7 with Protein phosphatase 1 catalytic subnit, alpha isoenzyme (PP1A) (Hs00267568_m1), β-2 microglobulin (B2M) (Hs00984230_m1) and glyceraldehydes 3-phosphate dehydrogenase (GAPDH) (Hs02786624_g1) as the housekeeping genes according to the manufacturer's instructions.

Western blot

Cell lysates were prepared by harvesting with protein extraction buffer (PEB) containing phosphatase and protease inhibitor cocktails (Sigma) as previously described[15]. Samples harvested with PEB were then subjected to SDS-PAGE with standard molecular weight ladder as a control after which they were blotted on a nitrocellulose membrane. Expression of collagen Iα1 was analyzed using a rabbit anti-human collagen I antibody (Abcam, ab34710), mouse anti-human non-muscle myosin IIB (Abcam, ab684) and anti-human β-tubulin (Abcam, ab24610) with a goat anti-rabbit IRDye 800 CW (LICOR, Lincoln, NE) as the secondary antibody after which visualization was done with the ODYSSEY infrared imaging system (LICOR). Signal from

detected bands were normalized to total protein using the REVERT total protein stain (LI-COR, Lincoln, NE, USA) according the manufacturer's instruction.

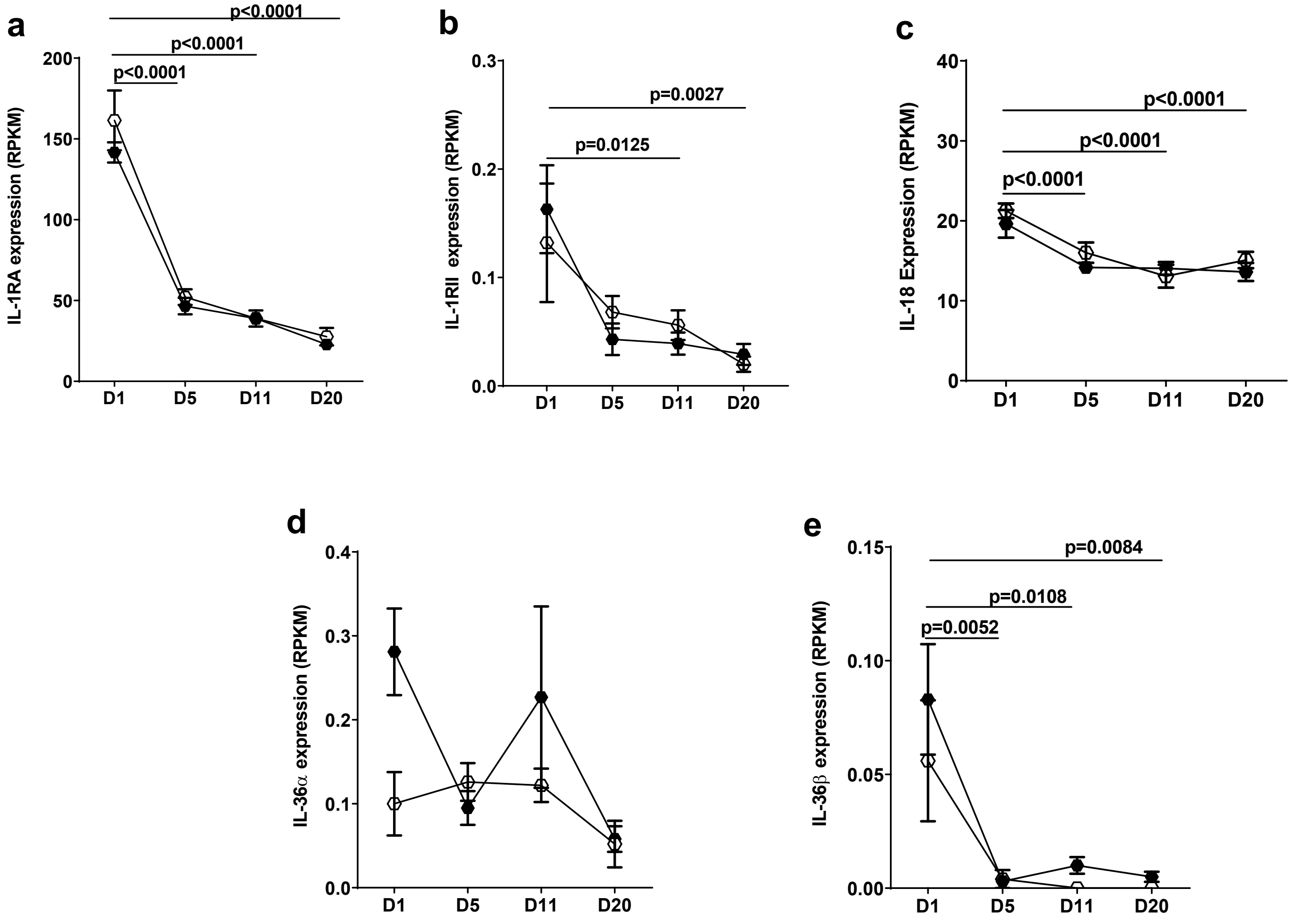

Figure S1. Expression of IL-1 family members (IL-1RA, IL-1RII, IL-18, IL-36α and IL-36β) in differentiated air-liquid interface (ALI) cultures of primary airway epithelial cells. Primary airway epithelial cells (PAECs) from non-asthmatics (n=5) and asthmatics (n=10) were cultured at an air-liquid interface, RNA and supernatants were collected at Days (D) 1, 5, 11 and 20. a) IL-1RA, b) IL-1RII, c) IL-18, d) IL-36α and e) IL-36β expression from PAECs are expressed as normalized to base pair reads. Mean±SEM for 5 Non-Asthmatics and 10 Asthmatics are shown.

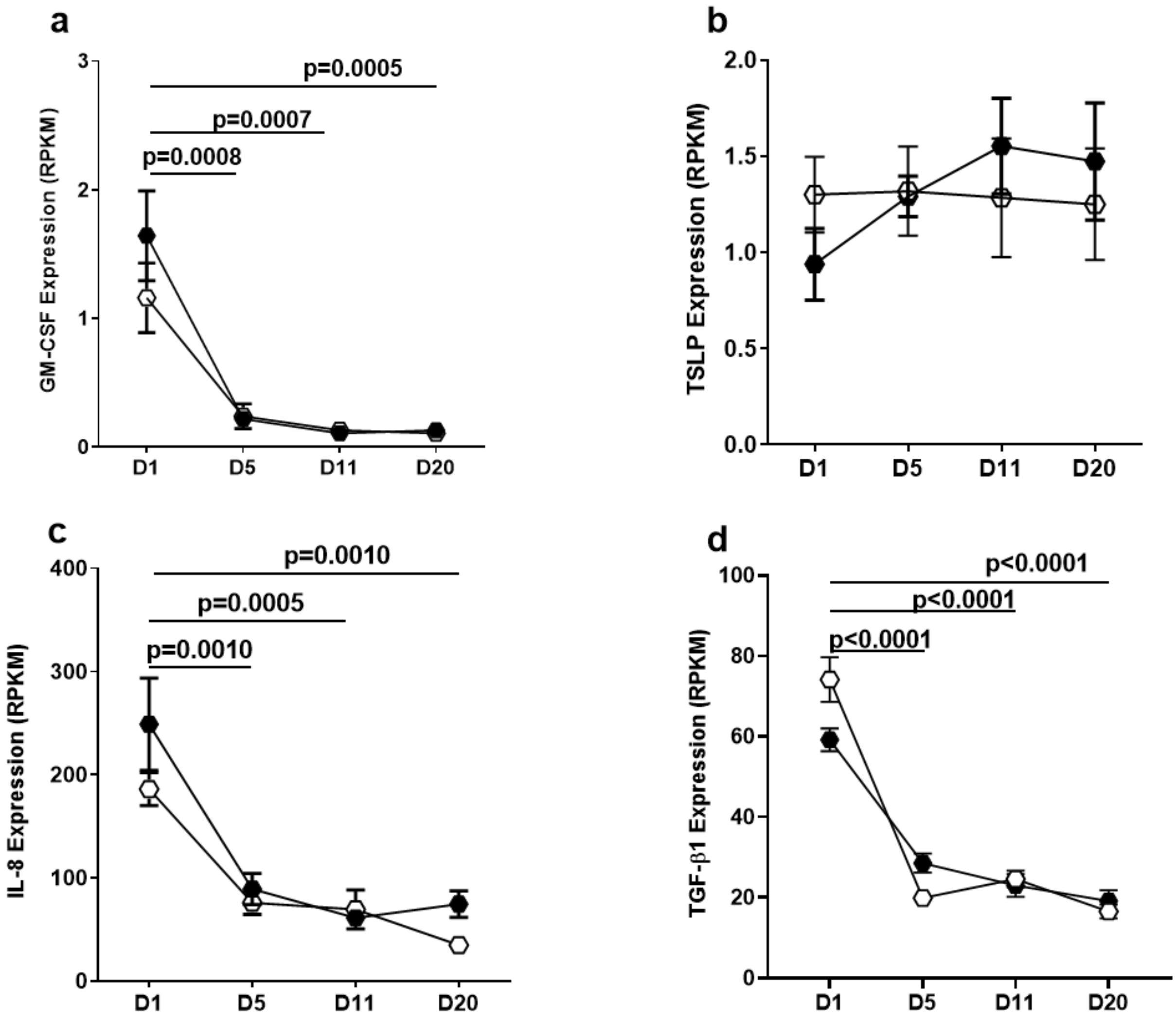

Figure S2. Expression of common epithelial-mediators involved in repair over time in differentiated air-liquid interface (ALI) cultures of primary airway epithelial cells. Primary airway epithelial cells (PAECs) from non-asthmatics (n=5) and asthmatics (n=10) were cultured at an air-liquid interface, RNA and supernatants were collected at Days (D) 1, 5, 11 and 20. a) GM-CSF, b) TSLP, c) IL-8 and d)TGF-β from PAECs are expressed as normalized to base pair reads. Means±SEM for 5 Non-Asthmatics and 10 Asthmatics are shown

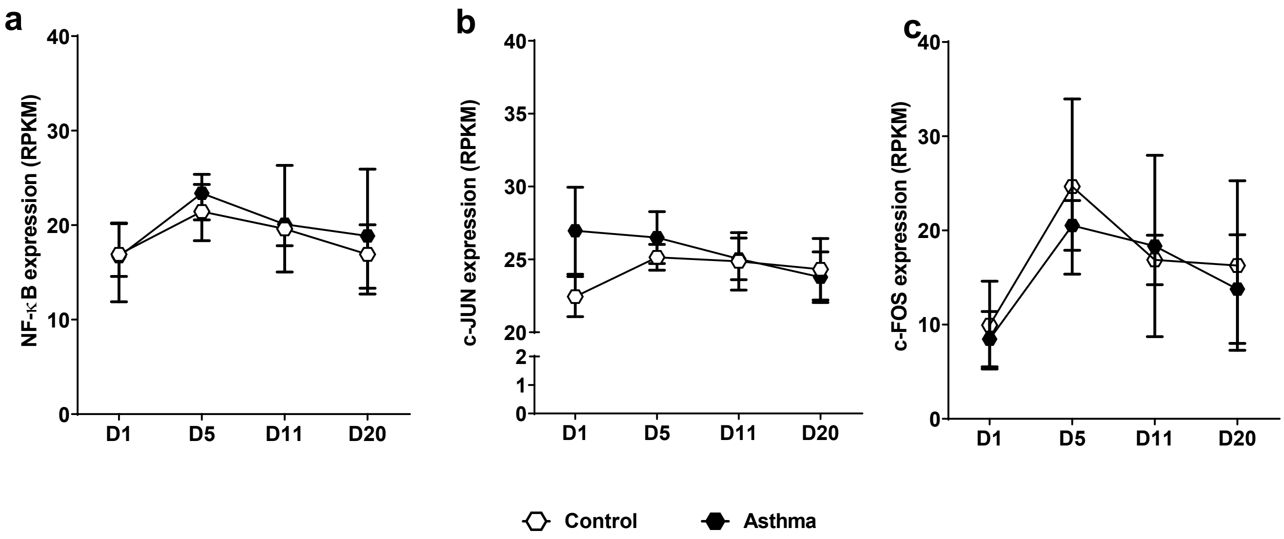

Figure S3. Expression of common transcription factors in differentiated air-liquid interface (ALI) cultures of primary airway epithelial cells that control IL-1 expression. Primary airway epithelial cells (PAECs) from non-asthmatics (n=5) and asthmatics (n=10) were cultured at an air-liquid interface, RNA and supernatants were collected at Days (D) 1, 5, 11 and 20. a) NF-κB, b) c-JUN and c) c-FOS expression from PAECs are expressed as normalized to base pair reads. Mean±SEM for 5 Non-Asthmatics and 10 Asthmatics are shown.

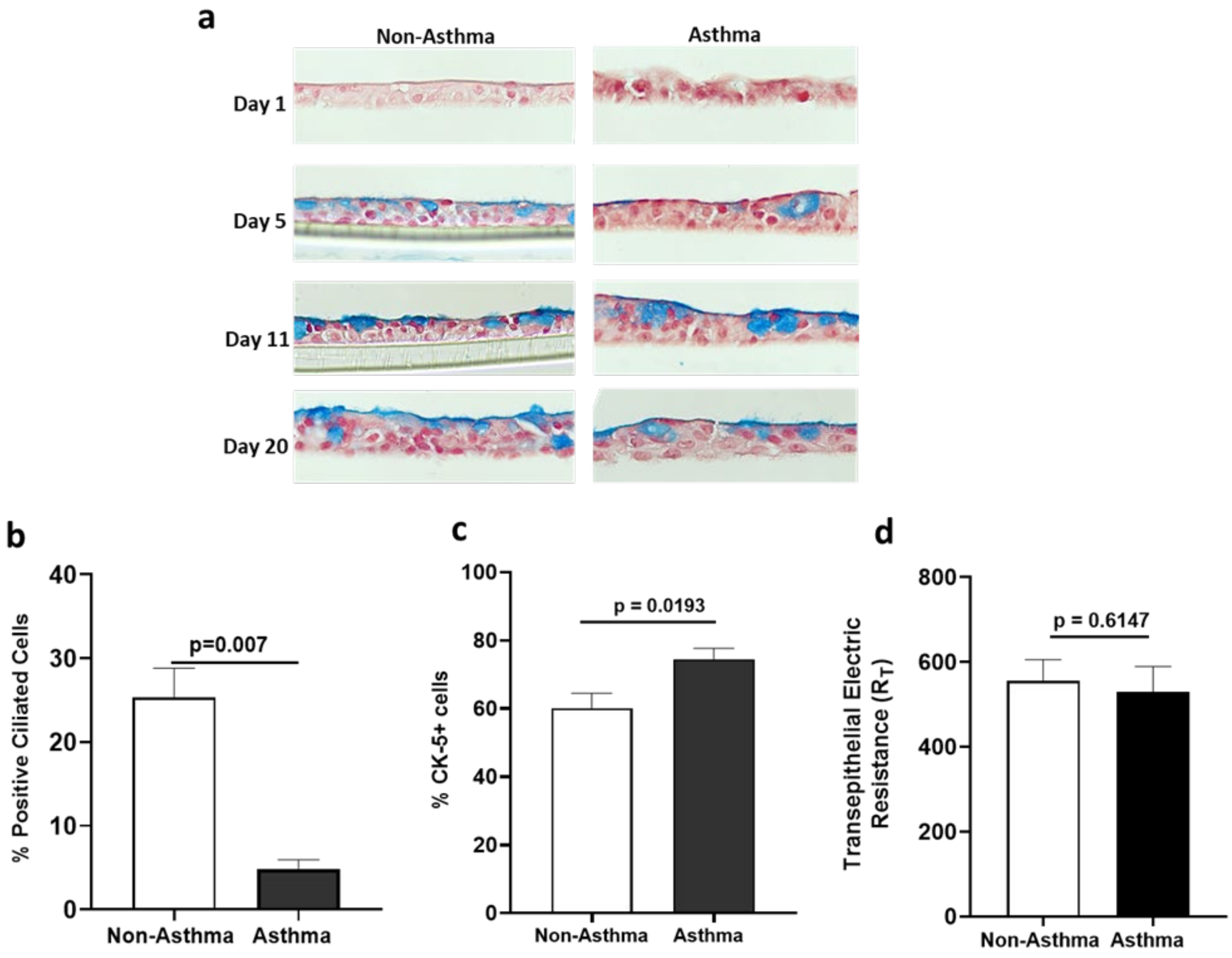

Figure S4. Muco-ciliary differentiation and transepithelial electric resistance for air-liquid interface (ALI) cultures of primary airway epithelial cells. Primary airway epithelial cells (PAECs) from non-asthmatics and asthmatics were cultured at an air-liquid interface. a) Representative images of Alcian blue stain for mucins, counterstained with neutral red at days (D) 1, 5, 11 and 20, b) Percentage ciliated cells at day 20 c) Percentage CK-5 positive cells at day 20 and d) transepithelial electric resistance comparing non-asthma and asthma at day 20. Means±SEM for 5 Non-Asthmatics and 10 Asthmatics are shown.

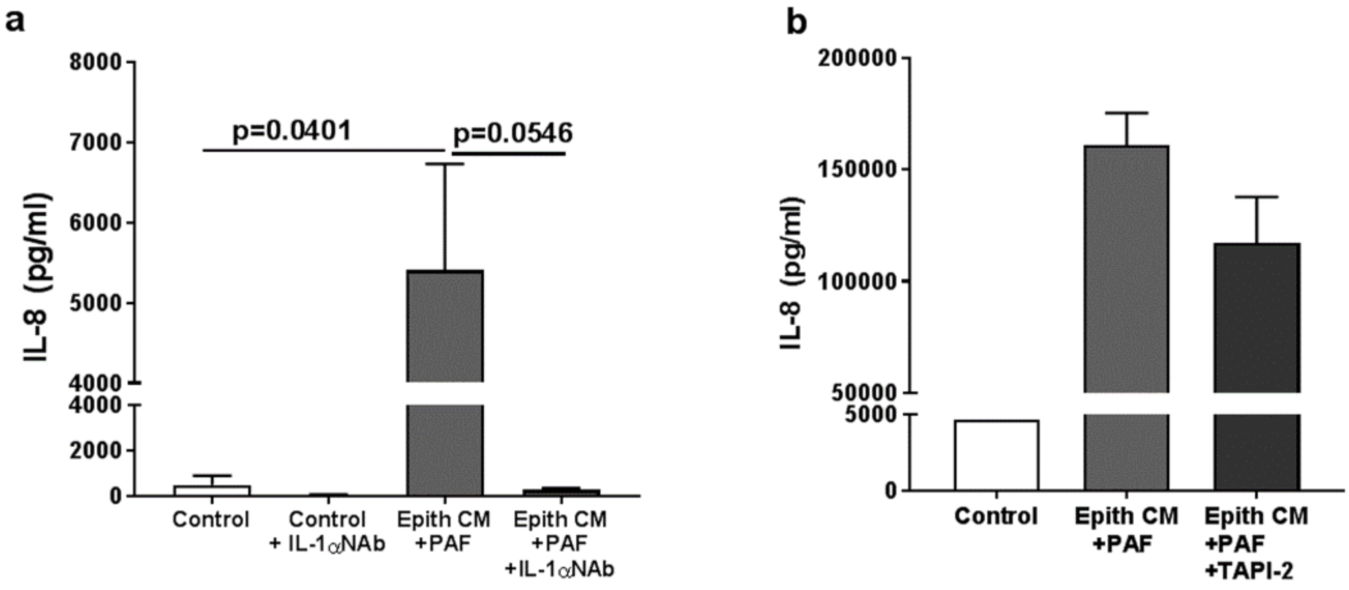

Figure S5. Epithelial-derived IL-1α is responsible for cross-talk between airway epithelium and airway fibroblasts. IL-8 concentration released from confluent non-asthmatic primary airway fibroblasts incubated without (Control) or with conditioned medium (CM) from confluent airway epithelial 16HBE14o- cells, in the presence and absence of a) 4 µg·/mL IL-1α neutralising antibody (NAb) and b) 10 µM of TAPI-2 acetate a broad matrix metalloproteinase inhibitor. Data is presented as mean ± SEM of 3 independent experiments. Exact P values indicated

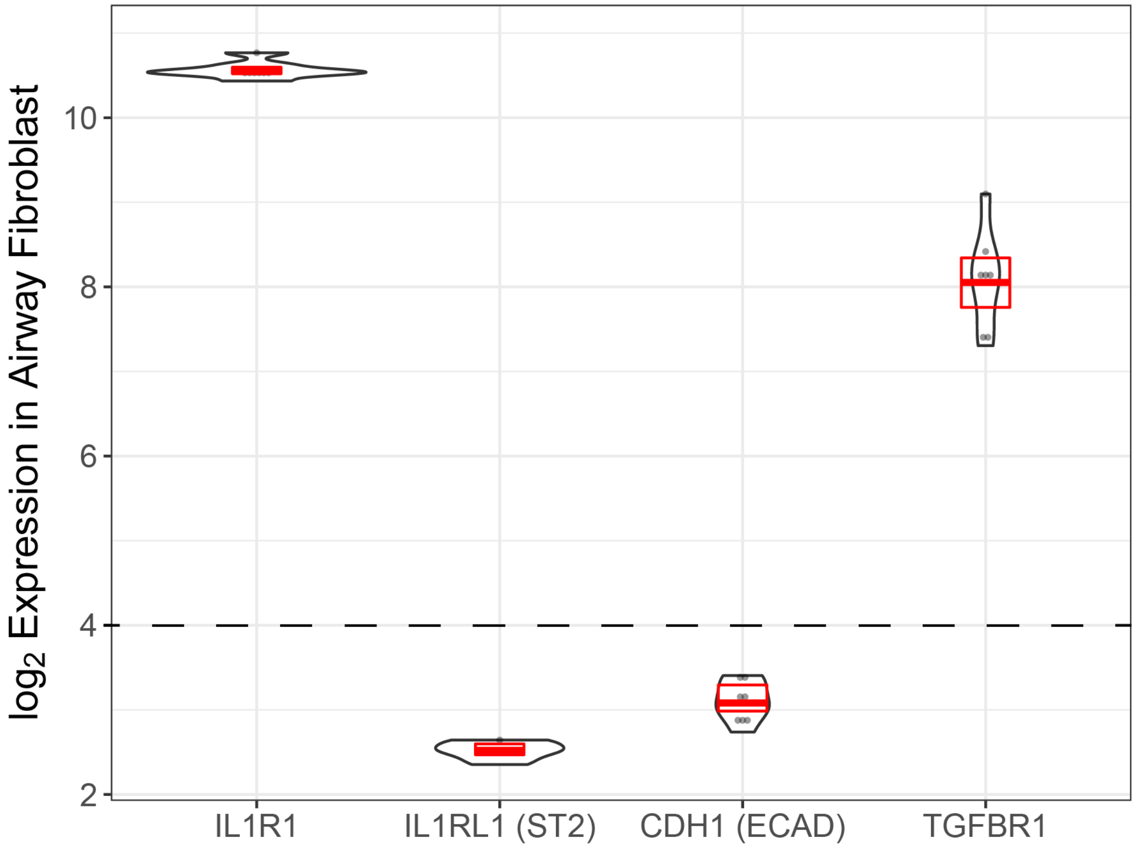

Figure S6. Receptor expression on airway fibroblasts. RNA from airway fibroblasts (n=18) was assessed for IL-1R1, the IL1RL1/ST2 receptor, CDH1 E-cadherin, and TGF-BR1. The dotted line indicates an arbitrary cut off for back-ground levels of transcript.

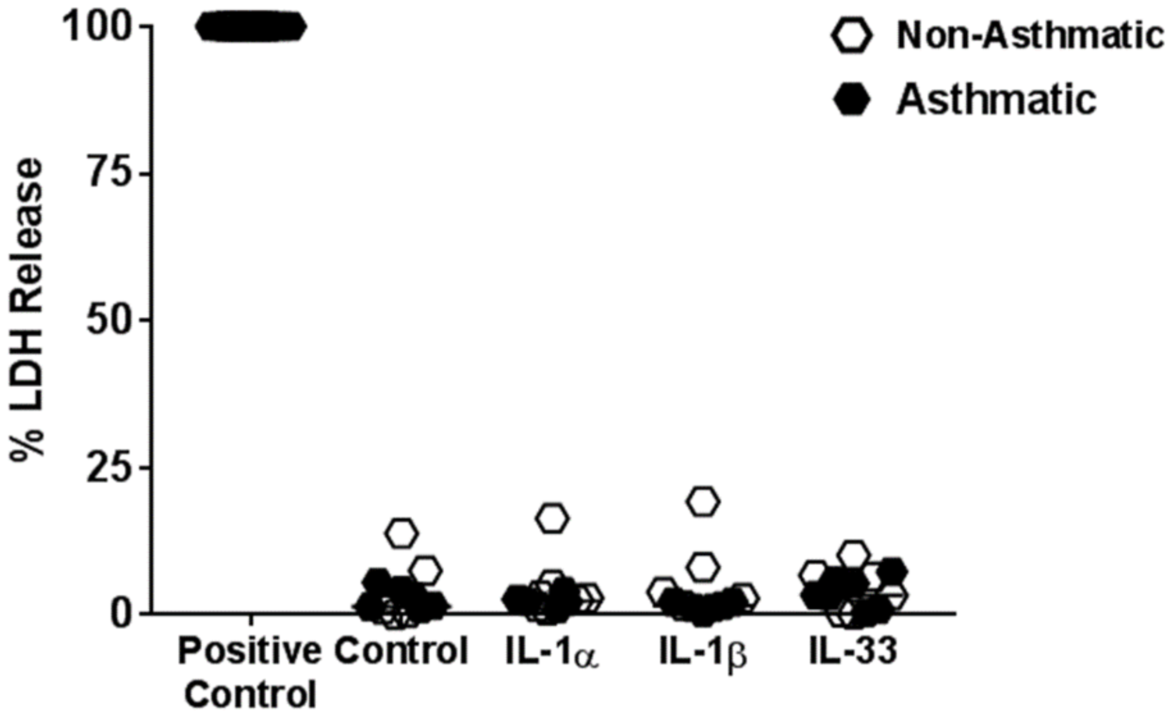

Figure S7. Assessment of cell death of primary airway fibroblasts after cytokine stimulation. Primary airway fibroblasts from non-asthmatics and asthmatics were grown to confluence on collagen I coated plates and stimulated with or without 1ng/ml recombinant human IL-1α, IL-1β or IL-33 for 24 hours. Percentage lactate dehydrogenase (LDH) released from cells after 24 hours was then assessed.

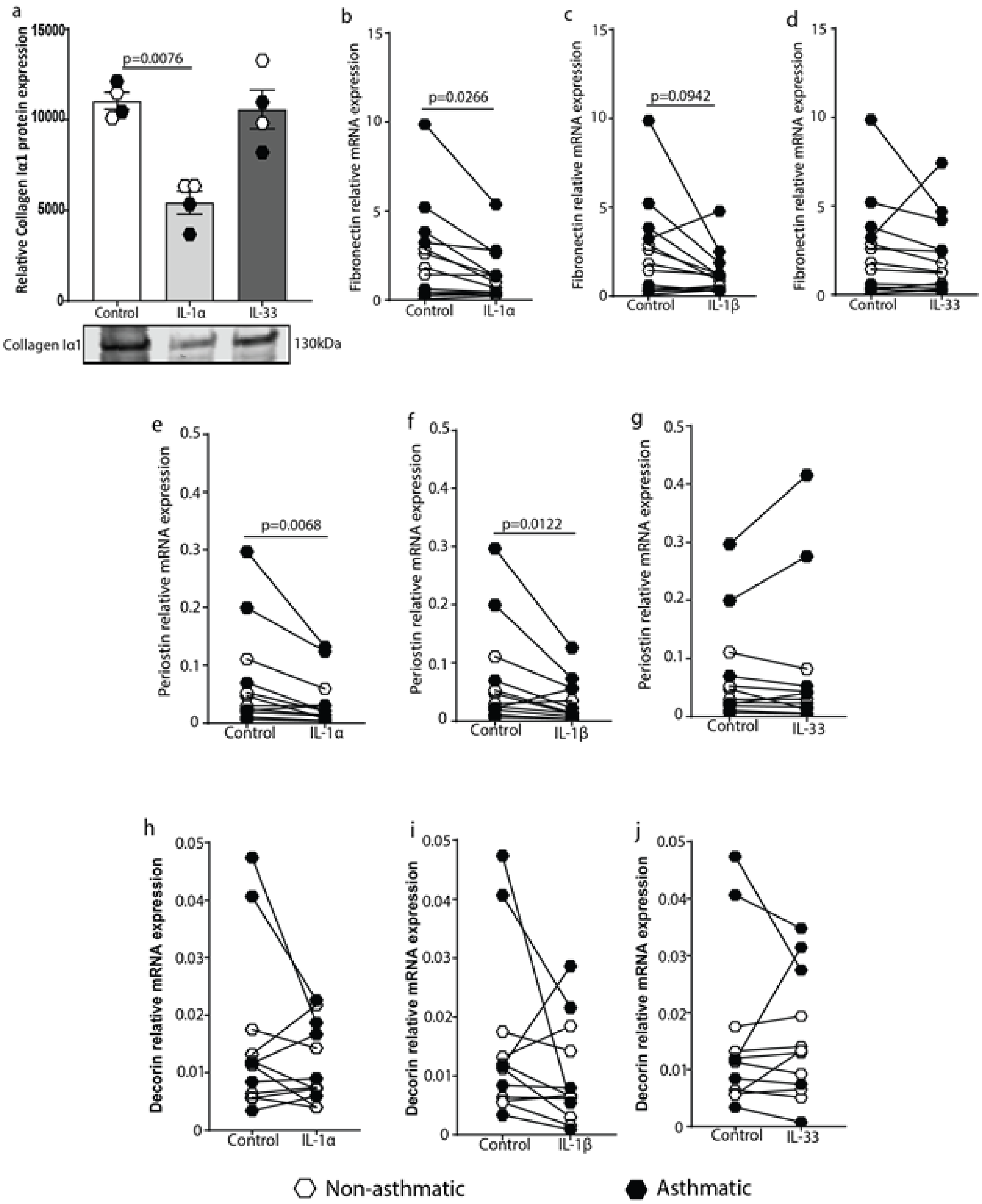

Figure S8. Decrease in ECM expression after IL-1α stimulation. Primary airway fibroblasts were grown to confluence on collagen I coated plates. a) Protein expression and representative blot images of Collagen Iα1 normalized to total protein. mRNA expression of (b-d) Fibronectin, (e-g) Periostin and (h-j) Decorin, after stimulation with or without 1ng/ml recombinant human IL-1α, IL-1β or IL-33 for 24 hours. Exact P values indicated.

a

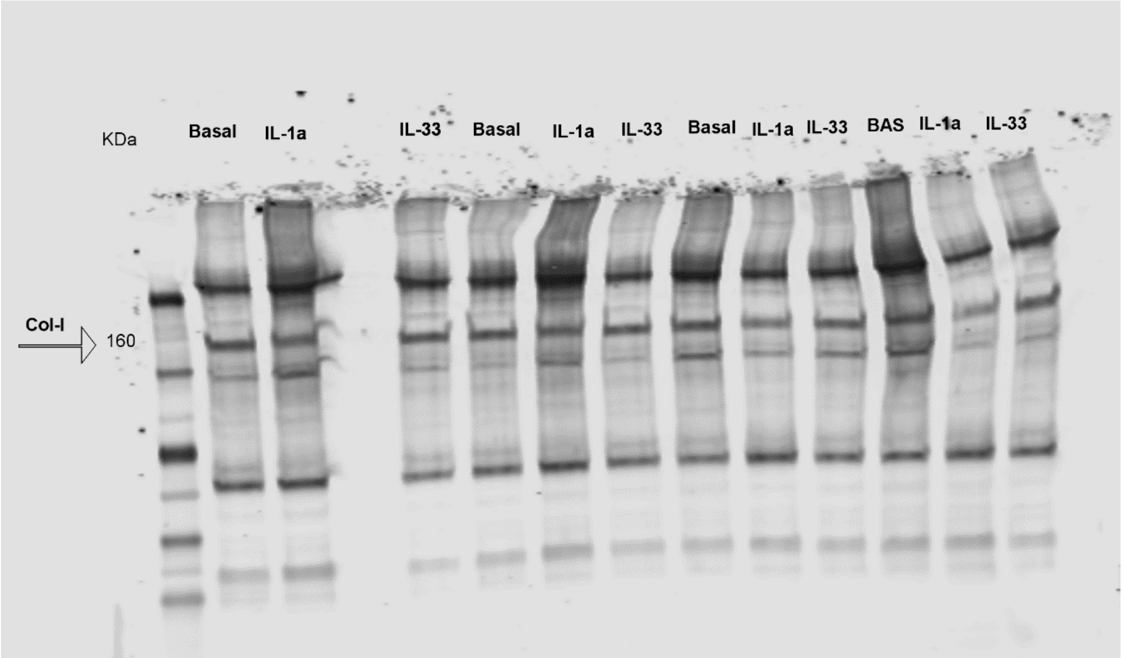

b

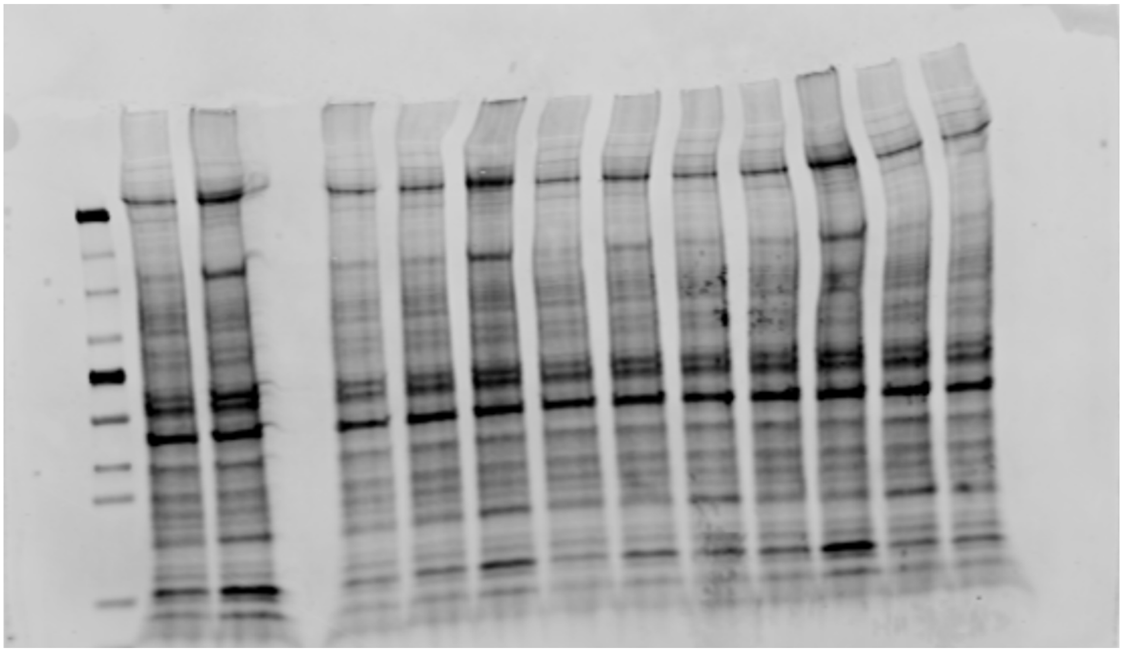

Figure S9. Representative western blot images for collagen Iα1. Primary airway fibroblasts were grown to confluence on collagen I coated plates and left unstimulated or stimulated with IL-1α and IL-33. a) Representative full blots containing the bands of collagen Iα1, b) Representative full blot of total protein bands used for normalization according the REVERT total protein stain protocol (LI-COR, Lincoln, NE, USA).

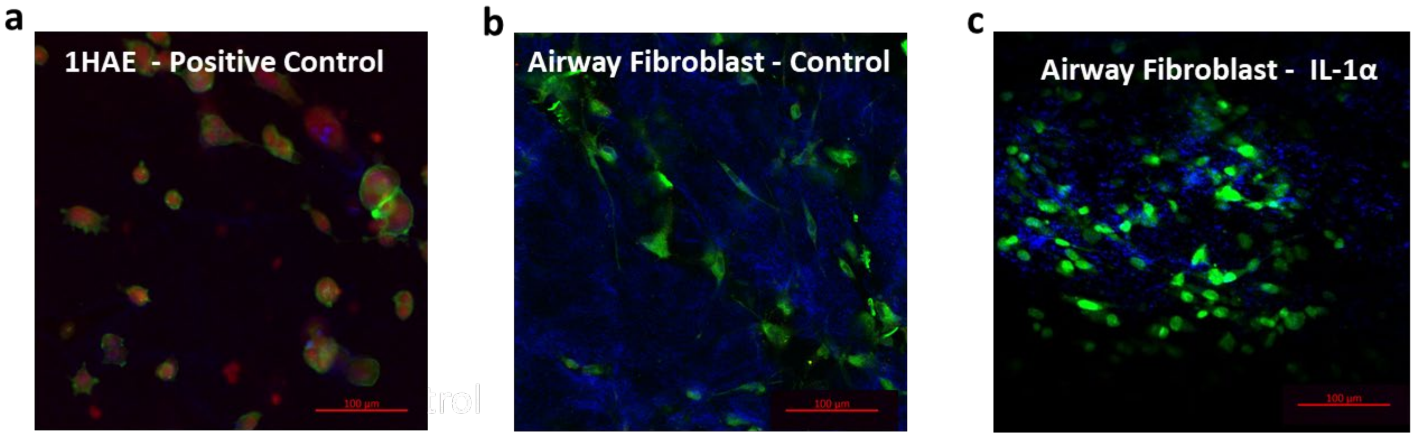

Figure S10. E-cadherin expression in primary airway fibroblasts. Representative images showing E-cadherin staining in red, Phalloidin staining for F-actin in green, and second-hamonic-signal for collagen in blue. a) The positive control gel contains the 1HAE airway epithelial cell line (n=3) which is positive for E-cadherin compared to no staining for E-cadherin in primary airway fibroblasts (PAF) treated with either b) media control and or c) 1ng/ml IL-1α-stimulation (n=5).

a

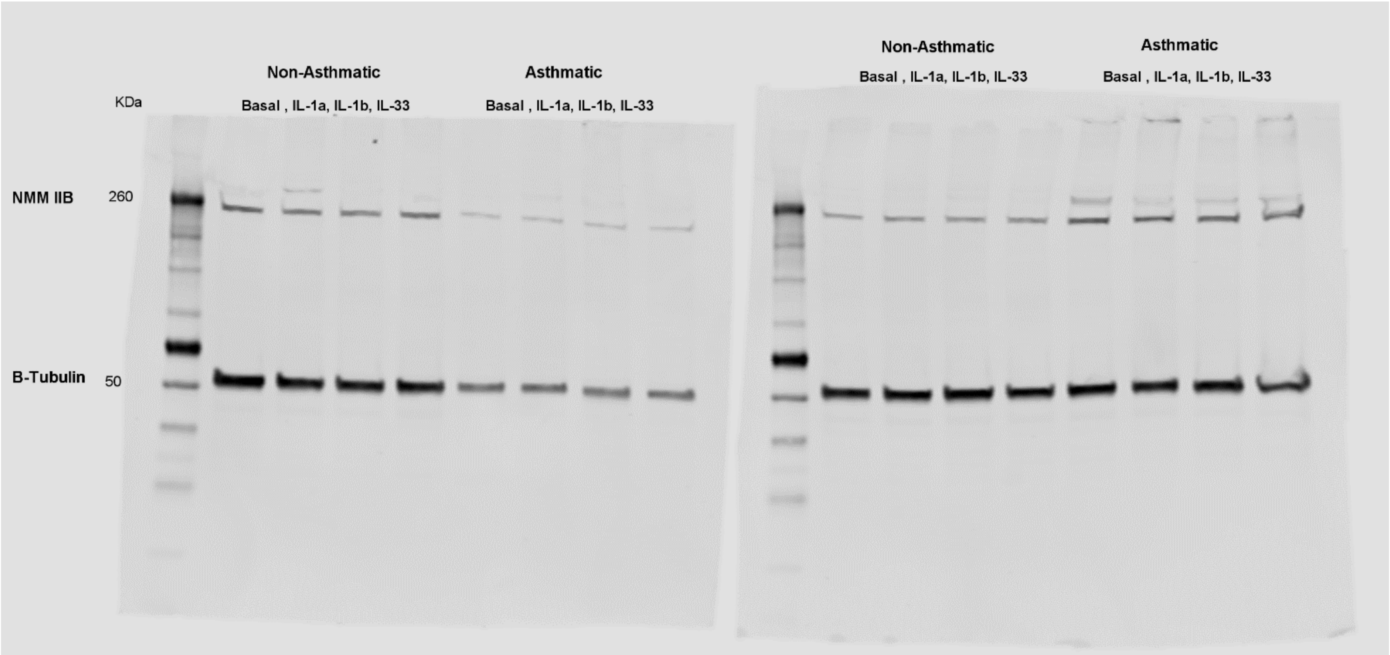

b

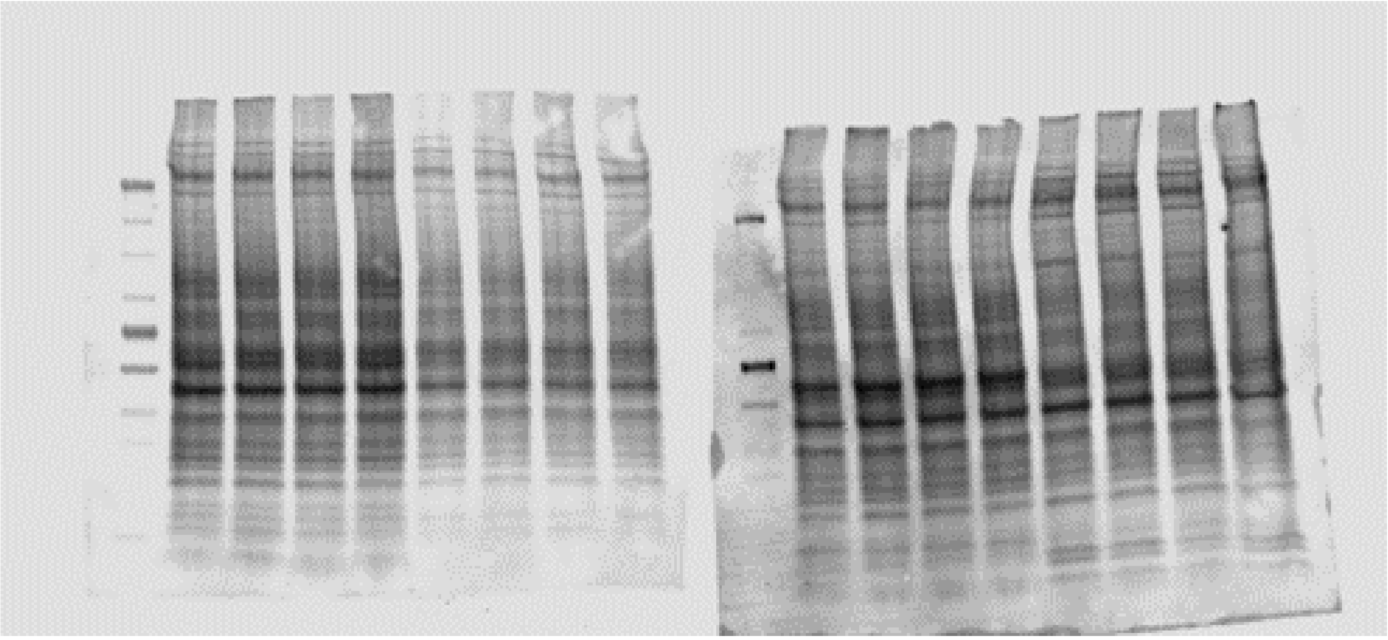

Figure S11. Representative western blot images for non-muscle myosin IIB and β-tubulin. Primary airway fibroblasts were grown to confluence on collagen I coated plates and left unstimulated or stimulated with IL-1α, IL-1β and IL-33. a) Representative full blots of non-muscle myosin IIB and β-Tubulin, b) Representative full blots of total protein bands used for normalization according the REVERT total protein stain protocol (LI-COR, Lincoln, NE, USA).

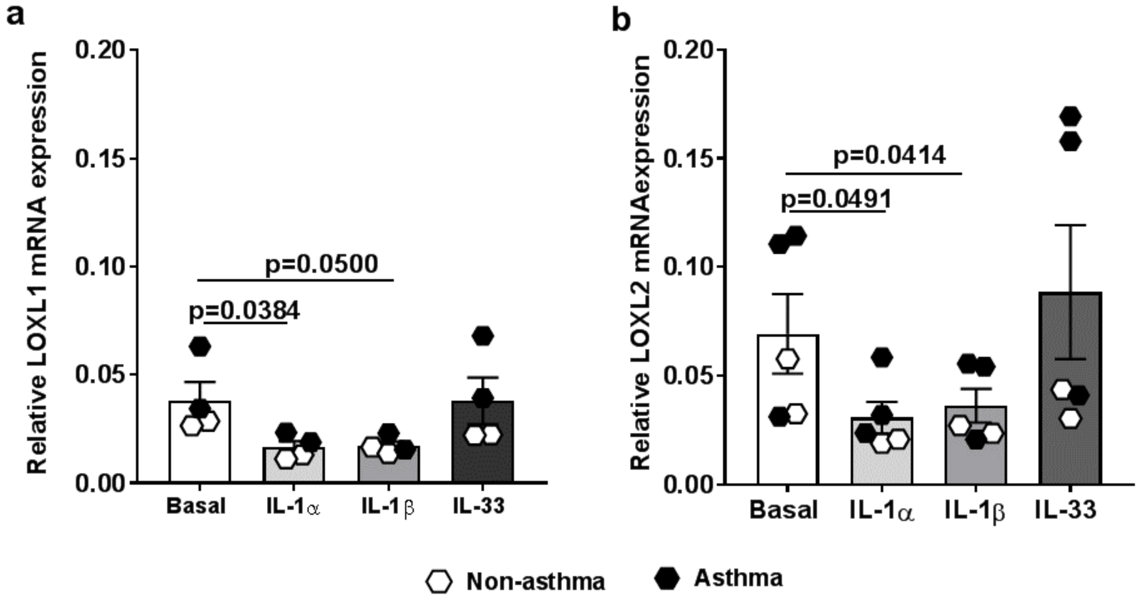

Figure S12. Effect of IL-1 stimulation on the expression of Lysyl oxidase-like (LOXL) 1 and 2 enzymes. Primary airway fibroblasts from non-asthmatics and asthmatics were grown to confluence on collagen I coated plates and stimulated with or without 1ng/ml recombinant human IL-1α, IL-1β or IL-33 for 24hours. Relative protein expression of a) LOXL1 and b) LOXL2 in primary airway fibroblasts after 24 hours. Exact P values indicated.

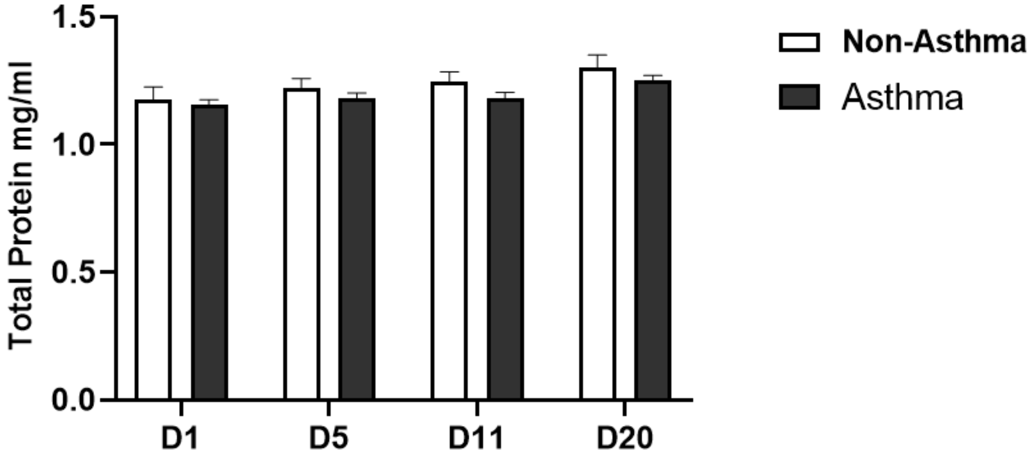

Figure S13. Total protein concentration in cell culture supernatant collected from ALI's. Primary airway epithelial cells (PAECs) from non-asthmatics (n=5) and asthmatics (n=10) were cultured at an air-liquid interface, supernatants were collected at Days (D) 0, 5, 11 and 20 and assess for total protein (mg/ml) using the Pierce Rapid Gold BCA Protein Assay Kit (ThermoFisher). Means ± SEM for 5 Non-Asthmatics and 10 Asthmatics are shown.

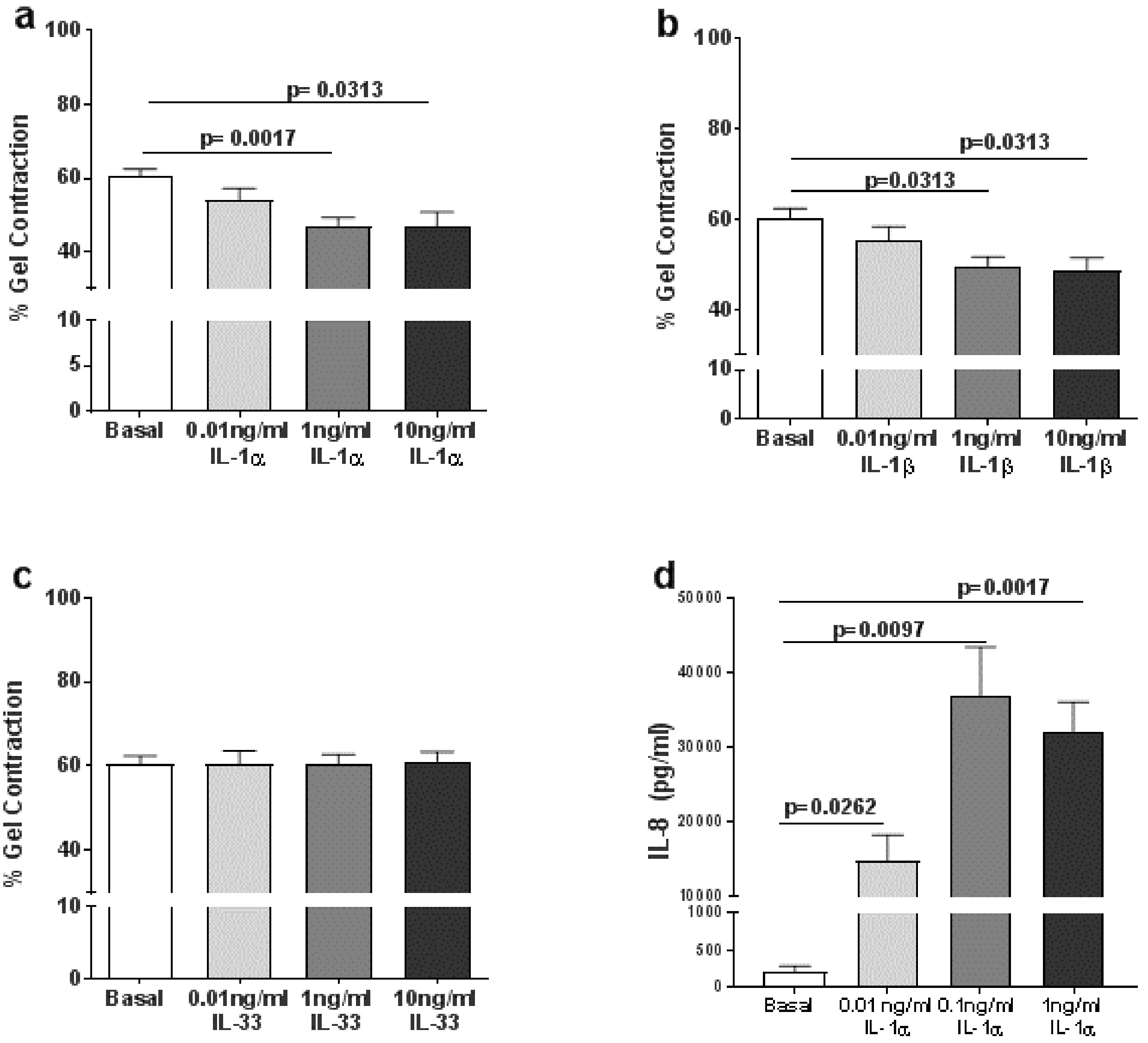

Figure S14. Dose effect of IL-1 and IL-33 stimulation on fibroblast collagen 1 gel contraction and IL-8 release. Non-asthmatic primary airway fibroblasts (PAFs) were grown to confluence and seeded in collagen I gels in the presence or absence of 0.1, 1 and 10 ng/ml IL-1α, IL-1β or IL-33 and allowed to contract for 24 hours. a) Percentage gel contraction comparing control PAF contraction rates after IL-1α stimulation b) Percentage gel contraction comparing control PAF contraction rates after IL-1β stimulation c) Percentage gel contraction comparing control PAF contraction rates after IL-33 stimulation. d) Primary airway fibroblasts from control individuals were also seeded on culture plates, grown to confluence and serum deprived overnight. CXCL8/IL-8 concentration released after fibroblasts after stimulation with IL-1α at concentrations 0.01, 0.1, 1ng/ml for 24 hours. Data is presented as Mean±SEM of 6 independent experiments. Exact P values indicated.

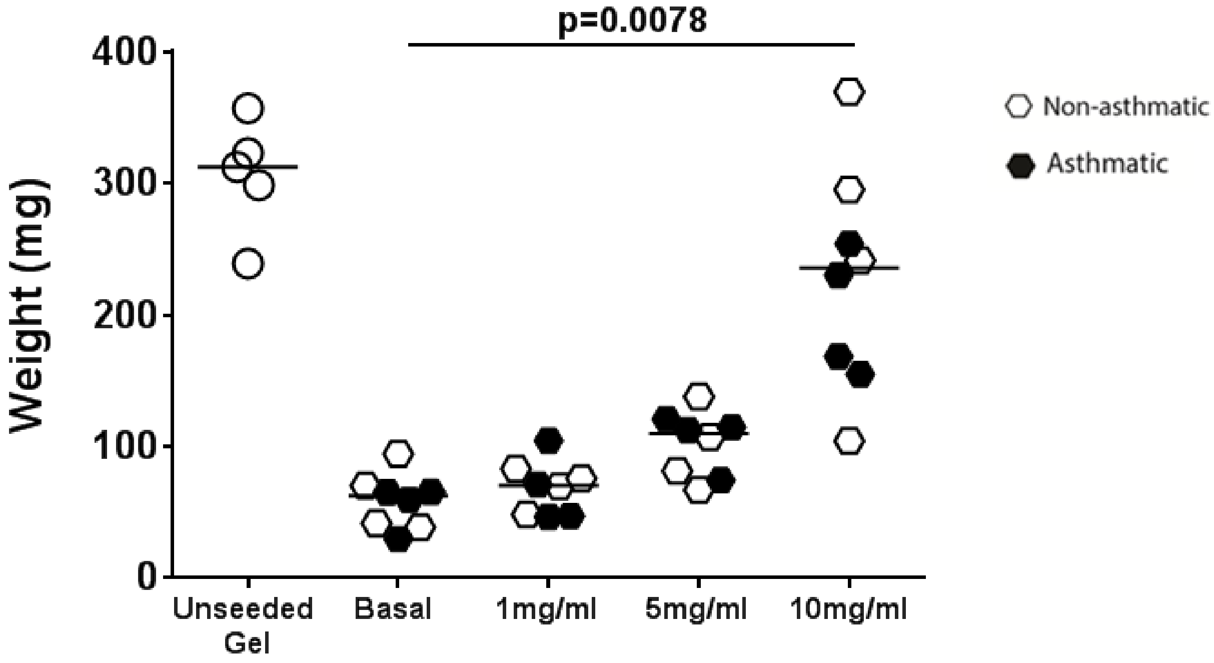

Figure S15. Dose effect of BAPN stimulation on fibroblast collagen 1 gel contraction. Primary airway fibroblasts (PAFs) were grown to confluence and seeded on collagen I gels in the presence or absence of 1, 5 and 10 mg/ml β-aminopropionitrile (BAPN) and allowed to contract for 24 hours. Gel weight after gel contraction comparing control PAF contraction BAPN stimulation. Exact P value indicated.

Online Supplementary REFERENCES

1 Pechkovsky, D. V. *et al.* Human lung parenchyma but not proximal bronchi produces fibroblasts with enhanced TGF-beta signaling and alpha-SMA expression. *Am J Respir Cell Mol Biol* **43**, 641-651, doi:10.1165/rcmb.2009-0318OC (2010).

2 Goodpaster, T. *et al.* An immunohistochemical method for identifying fibroblasts in formalin-fixed, paraffin-embedded tissue. *J Histochem Cytochem* **56**, 347-358, doi:10.1369/jhc.7A7287.2007 (2008).

3 Campbell, J. D. *et al.* A gene expression signature of emphysema-related lung destruction and its reversal by the tripeptide GHK. *Genome medicine* **4**, 67, doi:10.1186/gm367 [doi] (2012).

4 Mostaço-Guidolin, L. B. *et al.* Defective Fibrillar Collagen Organization by Fibroblasts Contributes to Airway Remodeling in Asthma. *Am J Respir Crit Care Med, [online ahead of print] 05 April 2019; https://www.atsjournals.org/doi/abs/10.1164/rccm.201810-1855OC.*, doi:10.1164/rccm.201810-1855OC.

5 Mostaço-Guidolin, L. B. *et al.* Collagen morphology and texture analysis: from statistics to classification. *Sci Rep* **3**, 2190, doi:10.1038/srep02190 (2013).

6 Steger, C. An Unbiased Detector of Curvilinear Structures. *IEEE Transactions on Pattern Analysis and Machine Intelligence* **20**, 113 - 125 (1998).

7 Zipfel, W. R., Williams, R. M. & Webb, W. W. Nonlinear magic: multiphoton microscopy in the biosciences. *Nat Biotechnol* **21**, 1369-1377, doi:10.1038/nbt899 (2003).

8 Xu, C., Zipfel, W., Shear, J. B., Williams, R. M. & Webb, W. W. Multiphoton fluorescence excitation: new spectral windows for biological nonlinear microscopy. *Proc Natl Acad Sci U S A* **93**, 10763-10768, doi:10.1073/pnas.93.20.10763 (1996).

9 Haralick RM, Shanmugam K & I, D. 3(6):610–621 (Systems, Man and Cybernetics, IEEE Transactions on, 1973).

10 Fabry, B. *et al.* Selected contribution: time course and heterogeneity of contractile responses in cultured human airway smooth muscle cells. *J Appl Physiol (1985)* **91**, 986-994 (2001).

11 Smith, P. G., Deng, L., Fredberg, J. J. & Maksym, G. N. Mechanical strain increases cell stiffness through cytoskeletal filament reorganization. *Am J Physiol Lung Cell Mol Physiol* **285**, L456-463, doi:10.1152/ajplung.00329.2002 (2003).

12 Mijailovich, S. M., Kojic, M., Zivkovic, M., Fabry, B. & Fredberg, J. J. A finite element model of cell deformation during magnetic bead twisting. *J Appl Physiol (1985)* **93**, 1429-1436, doi:10.1152/japplphysiol.00255.2002 (2002).

13 Kim, D. *et al.* TopHat2: accurate alignment of transcriptomes in the presence of insertions, deletions and gene fusions. *Genome Biol* **14**, R36, doi:10.1186/gb-2013-14-4-r36 (2013).

14 Quinlan, A. R. & Hall, I. M. BEDTools: a flexible suite of utilities for comparing genomic features. *Bioinformatics* **26**, 841-842, doi:10.1093/bioinformatics/btq033 (2010).

15 Leung, C., Shaheen, F., Bernatchez, P. & Hackett, T. L. Expression of myoferlin in human airway epithelium and its role in cell adhesion and zonula occludens-1 expression. *PLoS One* **7**, e40478, doi:10.1371/journal.pone.0040478 (2012).
